# Engineering Large‐Grain Metal Halide Perovskite Films via Chemical Vapor Deposition for High‐Performance, Ultralow‐Noise Perovskite Photodetectors

**DOI:** 10.1002/advs.77049

**Published:** 2026-08-10

**Authors:** Roel Vanden Brande, Sudipta Seth, Lei Gao, Bapi Pradhan, Dilkhush Khicher, Anastasiia Bazylevska, Wei Qu, Christian Sternemann, Hai Wang, Steven De Feyter, Weidong Xu, Fei Zhang, Mischa Bonn, Johan Hofkens, Elke Debroye

**Affiliations:** ^1^ Molecular Imaging and Photonics, Department of Chemistry KU Leuven Leuven Belgium; ^2^ Max‐Planck‐Institute for Polymer Research Mainz Germany; ^3^ Fakultät Physik/DELTA Technische Universität Dortmund Dortmund Germany; ^4^ Henan University Zhengzhou China

**Keywords:** charge carrier dynamics, chemical vapor deposition, perovskite photodetectors, stability, ultralow‐noise current

## Abstract

Metal halide perovskites have emerged as promising semiconductors for high‐performance photodetectors and X‐ray sensors due to their high‐Z composition, excellent charge transport, and intrinsic defect tolerance. However, scalable fabrication of thick, pinhole‐free films with controlled crystallinity and long‐term stability remains challenging, limiting their device performance. Here, we demonstrate the chemical vapor deposition of large‐grain, phase‐pure CsPbBr_3_ films with thicknesses of ∼3 µm and grain sizes up to 35 µm, an order of magnitude larger than those obtained by conventional spin coating. By optimizing deposition parameters and substrate selection, we achieve smooth, uniform films with prolonged carrier decay times and enhanced charge transport, as evidenced by time‐resolved photoluminescence and Drude‐like behavior in terahertz photoconductivity measurements. Photodetectors based on these films exhibit ultralow dark currents (<1 pA cm^−^^2^ at 0 V), high switching ratios (∼10^4^), responsivities up to 0.176 A W^−^^1^, and detectivities of 8.4 × 10^1^ ^2^ Jones, while maintaining exceptional operational and ambient stability over one year. X‐ray sensitivity measurements further confirm the potential of these devices for low‐dose detection applications. This work establishes CVD as a reproducible, solvent‐free route to high‐quality halide perovskite films, providing a clear pathway to next‐generation photodetectors and X‐ray sensors with superior performance and long‐term stability.

## Graphical abstract

Chemical vapor deposition produces large‐grain, phase‐pure CsPbBr_3_ films (grains up to 35 µm) with Drude‐like terahertz photoconductivity and twofold higher carrier mobility versus spin‐coated references. These microstructural advances translate into photodetectors with a detectivity of 8.4 × 10^1^ ^2^ Jones, dark current below 1 pA cm^−^^2^, X‐ray sensitivity of 1.05 µC Gy^−^^1^ cm^−^^2^, and one‐year ambient stability.

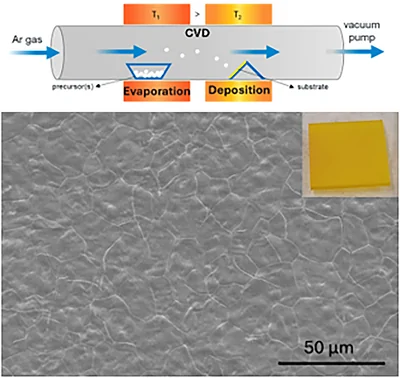

## 1 Introduction

Over the past decade, metal halide perovskites (MHPs) have emerged as a versatile class of semiconductor materials for optoelectronic applications. Beyond their record‐breaking performance in photovoltaics and light‐emitting diodes (LEDs), MHPs have recently shown great promise for photodetection and X‐ray detection technologies. Their unique combination of properties, including high atomic number (high‐Z) elements for efficient absorption of energetic X‐ray photons, excellent charge‐carrier generation and transport, low dark current, and an intrinsic defect‐tolerant nature, makes them ideal candidates for high‐performance photodetectors and highly sensitive X‐ray detectors. Together, these characteristics allow devices to operate at reduced radiation doses, which is important in minimizing radiation‐induced damage and relevant safety issues for their potential medical applications. Furthermore, compared to amorphous selenium (a‐Se), the current industry standard in X‐ray detection, lead‐based MHPs offer not only superior X‐ray sensitivity but also lower cost and more convenient fabrication [1, 2, 3, 4, 5].

While single‐crystal MHPs deliver exceptional optoelectronic performance [6], the fabrication of large‐area, pinhole‐free films with controlled thickness remains essential for practical photodetector and X‐ray detector applications [7, 8]. Although solution‐based techniques such as spin coating are widely used and have enabled perovskite solar cells to achieve power conversion efficiencies above 25%, these methods generally produce relatively thin polycrystalline layers. Such thin films are suitable for photovoltaic operation but are not optimized for applications that require micrometre‐scale thicknesses to ensure efficient light absorption or effective X‐ray attenuation. When extended to thicker films, solution processing often leads to inhomogeneous crystallization, pinhole formation, and increased defect densities [9, 10, 11, 12]. These defects act as charge‐carrier traps, limiting device efficiency and accelerating degradation via ion migration under environmental stressors such as light, air, and moisture. The key challenge, therefore, is to develop deposition methods that produce smooth, uniform, pinhole‐free perovskite layers with minimal defect density while retaining the superior properties of single crystals [13, 14, 15, 16, 17].

Chemical vapor deposition (CVD) offers an excellent solution to this challenge. Widely adopted in the semiconductor industry, CVD enables the fabrication of uniform, high‐purity films with excellent reproducibility and scalability [18, 19, 20]. Importantly, CVD is a solvent‐free technique, avoiding the issues associated with residual solvents in solution‐processed films, such as stoichiometric imbalance, crystallographic defects, and environmental concerns [20, 21, 22, 23]. In contrast to physical vapor deposition (PVD) methods, which often require high vacuum and can struggle to uniformly coat large areas or maintain consistent film thickness over extended lengths, CVD delivers highly conformal, thickness‐controlled films over large substrates with excellent uniformity [24]. This capability makes CVD particularly attractive for scalable fabrication of high‐performance perovskite layers and large‐area optoelectronic devices [25]. Despite the growing interest in CsPbBr_3_‐based photodetectors and X‐ray detectors, prior reports, whether based on solution processing or vapor‐phase growth, typically optimize only a subset of relevant material and device metrics [20, 23, 26, 27, 28].

Despite recent progress in the vapor‐phase growth of metal halide perovskites, establishing clear relationships between deposition conditions, microstructure evolution, charge‐carrier transport, and device performance remains a major challenge. Vapor‐phase processing is still considerably less mature than solution‐based approaches, and CVD in particular has not yet emerged as a widely adopted route for perovskite thin‐film fabrication due to the difficulty of controlling film microstructure and linking growth parameters to optoelectronic functionality [29]. These challenges become especially critical for photodetection and X‐ray sensing applications, where micrometre‐thick absorber layers require the simultaneous optimization of crystallinity, grain‐boundary density, carrier transport, leakage‐current suppression, and long‐term operational stability. While previous studies on CVD‐grown CsPbBr_3_ have largely focused on morphological optimization or the improvement of individual device metrics, a comprehensive understanding of how CVD growth conditions govern charge transport and detector operation remains lacking. Here, we address this gap by establishing direct structure–transport–device correlations in micrometre‐thick CsPbBr_3_ films through a combination of microstructural analysis, time‐resolved photoluminescence, terahertz photoconductivity, and comprehensive device and noise characterization. This approach provides mechanistic insight into the influence of CVD‐induced microstructural evolution on charge transport and detector performance, thereby offering design principles for vapor‐grown perovskite radiation detectors. The principal bottlenecks in CVD growth of thick CsPbBr_3_ films are: (i) maintaining a stoichiometric vapor phase despite the markedly different volatilities of CsBr and PbBr_2_; (ii) controlling nucleation density and supersaturation to favor large, uniform grains over grain‐boundary‐dominated microstructures; and (iii) suppressing secondary‐phase formation (CsPb_2_Br_5_, Cs_4_PbBr_6_). The present work addresses each of these challenges directly through systematic optimization of precursor ratios, reaction time, and substrate positioning.

In this work, we demonstrate the reproducible growth of highly crystalline CsPbBr_3_ thin films using CVD. The resulting ∼3 µm‐thick films exhibit grain sizes up to 35 µm, which are an order of magnitude larger than those obtained via spin coating, along with smooth, pinhole‐free morphologies and prolonged carrier decay times. These material advances translate into photodetectors with ultralow dark currents, high switching ratios (>10^4^), responsivities up to 0.176 A·W^−^^1^, and detectivities of 8.4 × 10^1^ ^2^ Jones. Furthermore, the devices maintain excellent structural and operational stability for over a year under ambient conditions. By establishing a clear link between controlled film growth, microstructure, and device performance, our study highlights CVD as a key enabler for next‐generation perovskite‐based photodetectors and X‐ray sensors. This study therefore goes beyond incremental improvements in individual metrics and instead highlights the importance of achieving a synergistic combination of structural, transport, noise, and stability characteristics through reproducible vapor‐phase growth amenable to scale‐up.

## 2 Experimental Section

### 2.1 Chemicals

Cesium bromide (99.9%) and lead bromide (99.999%) were purchased from Thermo Fisher to synthesize CsPbBr_3_ films. For spin coating, dimethyl sulfoxide (DMSO, 99.9%) and chlorobenzene (99%) were purchased from Merck Life Science and Acros Organics, respectively.

### 2.2 CVD of the CsPbBr_3_ Films

CsPbBr_3_ films were synthesized using a custom‐built CVD system consisting of two independently movable tube furnaces, a quartz tube reactor (25 cm diameter), and a gas flow controller. In a typical procedure, CsBr (66 mg, 0.31 mmol) and PbBr_2_ (59 mg, 0.16 mmol) are ground and then loaded into a quartz boat. The precursor‐loaded boat is placed in the first heating zone (oven 1), while the substrates are positioned downstream in the second zone (oven 2), mounted on a substrate holder with an inclination of 30°. Fused silica and soda‐lime glass substrates measuring 1.8 × 1.8 cm^2^ were used for all depositions. The deposition strategy can be extended to larger‐area substrates upon using larger‐diameter tube reactors and slightly modifying the CVD geometry. The reactor is evacuated to 10^−^^3^ mbar and purged three times with argon to remove residual oxygen and moisture. During deposition, the precursor zone is heated to 530°C to induce evaporation, while the substrate zone is maintained at 230°C. A continuous argon flow of 165 nccm ensures transport of the vapor‐phase species to the growth region. After the desired reaction time (60 min), oven 1 is retracted to terminate the precursor supply, and oven 2 is allowed to cool naturally to room temperature. Additional practical considerations regarding CVD growth and common failure modes are summarized in Table S1.

### 2.3 Spin Coating of CsPbBr_3_ thin Films

The synthesis of CsPbBr_3_ films was performed following a previously reported procedure [30]. The precursor solution was prepared by dissolving CsBr (0.3 mmol) and PbBr_2_ (0.3 mmol) in 1 mL of dimethyl sulfoxide (DMSO) at 60°C, followed by stirring for 30 min. The solution was then left to stir at room temperature for 16 h. Glass substrates were cleaned by sequential sonication in Hellmanex solution, deionized water, acetone, and isopropanol for 15 min each, and subsequently treated with UV‐ozone for 60 min. The substrates were then transferred to a nitrogen‐filled glovebox and spin‐coated with 60 µL of CsPbBr_3_ solution using a one‐step process (1200 rpm for 15 s, followed by 3000 rpm for 75 s). To induce rapid crystallization and remove residual DMSO, 1 mL of chlorobenzene was dripped onto the spinning substrate 30 s before the end of the second spin step. Finally, the films were annealed at 70°C for 30 min.

## 3 Results and Discussion

CVD is a widely used technique for synthesizing high‐quality, uniform crystalline films; however, its application in perovskite synthesis remains relatively new [19, 20, 31]. Achieving high‐quality perovskite films via CVD requires a thorough understanding of key process parameters, including precursor evaporation temperature, nucleation temperature, gas flow rate, pressure, and substrate positioning [32, 33, 34, 35]. These parameters not only govern mass transport and film growth dynamics but also interact with one another. For instance, increasing gas flow can raise system pressure. Consequently, optimizing these conditions through an iterative approach is essential to enhancing the quality (film growth and morphology) and performance of the final perovskite layer. To support reproducible film preparation, we have catalogued the most common failure modes encountered during CVD growth, together with their likely physical origin and the corresponding mitigation strategies, in Table S1. In this study, we examine the effects of reaction time and substrate choice on the quality of CsPbBr_3_ films. Furthermore, we compare the photophysical characteristics and photodetector performance of the CVD‐deposited films with those produced via conventional spin coating to demonstrate the superiority of the CVD approach for the application of perovskites in future optoelectronics.

### 3.1 Optimizing CVD Conditions for High‐Quality CsPbBr_3_ Films

To ensure simultaneous evaporation, it is critical to maintain a stoichiometric vapor phase during gas transport and deposition. The temperature gradient between the two furnace zones is crucial for this balance, as it determines the relative vapor pressures and transport dynamics of CsBr and PbBr_2_. A controlled gradient ensures that both precursors evaporate at suitable rates in the upstream zone and that their vapor reaches the substrate in the correct stoichiometric ratio. If the gradient is too small, CsBr, with the higher boiling point, may not evaporate sufficiently, leading to a Pb‐rich vapor and nonstoichiometric film growth. Conversely, if the gradient is too steep, rapid downstream cooling can cause early condensation of CsBr vapor before it reaches the substrate, resulting in Cs‐rich deposits upstream and a Pb‐rich film on the substrate. Given the high boiling points of CsBr (∼1300°C) and PbBr_2_ (∼916°C), it remains uncertain whether such an ideal vapor‐phase balance is fully achieved. The precursors were pre‐ground together in a Cs:Pb molar ratio of 1.9:1 until a uniform yellow powder was obtained. The excess CsBr compensates for its lower volatility and reduced migration rate compared to PbBr_2_, ensuring a more balanced stoichiometry in the vapor phase above the substrate. As can be observed from the X‐ray detection (XRD) pattern of the ground precursor mixture (Figure S1), this grinding process already induces a partial reaction yielding an incomplete conversion to Cs‐Pb‐Br phases [36]. More broadly, these results illustrate a general principle for CVD of halide perovskite materials: phase purity is governed less by the nominal precursor stoichiometry than by the coupled control of differential evaporation, vapor transport, and local supersaturation at the substrate surface. The most common failure modes encountered during CVD growth, together with their likely physical origins and corresponding mitigation strategies, are catalogued in Table S1 to support reproducible film preparation and to serve as transferable guidance for CVD growth of other halide perovskite compositions.

The ground precursor powder is loaded into the CVD setup, which is evacuated to 10^−3 ^mbar. Then, the precursor zone (oven 1) is heated to 530°C, while the substrate zone (oven 2) is maintained at 230°C. After merely 30 min at a continuous argon flow of 165 nccm, a uniform film with a pinhole‐free morphology is obtained (Figure 1A). However, it is not phase‐pure, as XRD analysis reveals a Cs‐deficient side‐phase (CsPb_2_Br_5_) (Figure 1B). As mentioned earlier, the less volatile CsBr does not fully migrate within this time window, leaving a small amount of CsBr in the quartz boat. To avoid a stoichiometric imbalance, the reaction time was extended to 60 min. XRD analysis (Figure 1B) confirms the required stoichiometric balance, yielding phase‐pure CsPbBr_3_ films free of secondary phases such as CsPb_2_Br_5_ or Cs_4_PbBr_6_ under this prolonged reaction time [37, 38]. Profilometer scans indicated an average thickness of ∼2.9 ± 0.1 µm (Figure 1G), and scanning electron microscopy (SEM) displayed large grain sizes with an average of 13 µm, even reaching 25 µm (Figure 1C,D), though, compared to the 30 min reaction time, grain uniformity slightly decreased, and more pinholes appeared.

**FIGURE 1:**
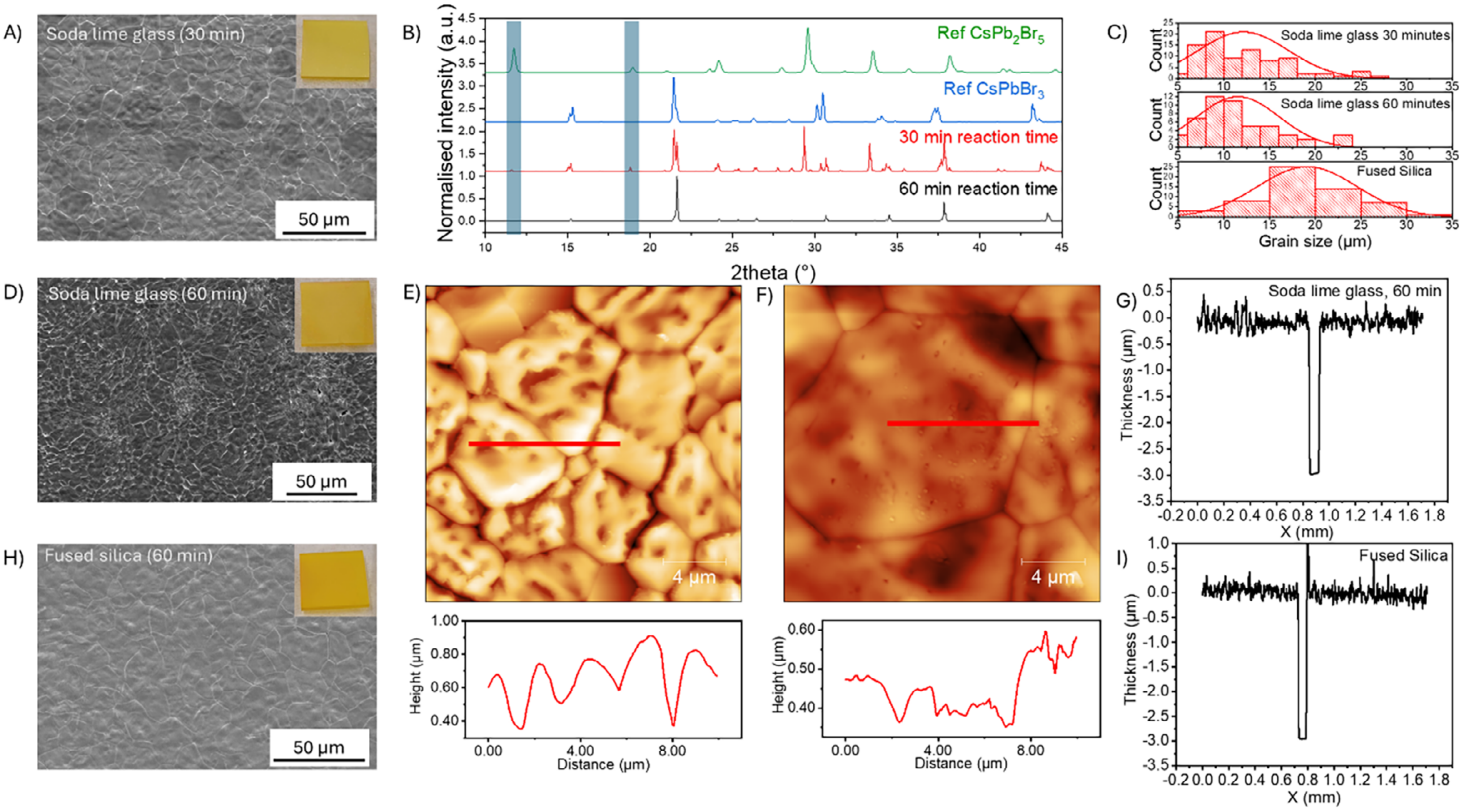
Film morphology, structure, and thickness. (A) SEM image of the CsPbBr_3_ film after 30 min of reaction time. (B) Comparison of the XRD patterns of the samples grown on soda lime glass. (C) Comparison of the histograms of the grain size distribution. (D, E, G) SEM, AFM image, and profilometer scan of CsPbBr_3_ film deposited after 60 min of reaction time on soda lime glass. (H, F, I) SEM, AFM image, and profilometer scan of CsPbBr_3_ film deposited on fused silica after 60 min of reaction time.

It should be noted that by slightly changing the parameters, both the presence and amount of the secondary phases can be controlled. While shorter reaction time promotes the presence of the CsPb_2_Br_5_ secondary phase. This can be explained by the high‐temperature instability of CsPb_2_Br_5_ and the volatile nature of PbBr_2_, which at longer reaction times promotes the conversion of CsPb_2_Br_5_ into CsPbBr_3_ and PbBr_2_ [39]. Conversely, positioning the substrate at the beginning of the second heating zone (oven 2), where the temperature gradient is maximal, induces a Cs‐rich growth environment, leading to the formation of the Cs_4_PbBr_6_ secondary phase (position 1 in Figure S2D). Figure S2 confirms the XRD patterns of the dual‐phase films. Together, these observations demonstrate that substrate positioning within the temperature gradient constitutes an additional independent handle for phase‐selective growth, complementing precursor ratio and reaction time as the primary control parameters.

### 3.2 Impact of Substrate on Film Structural Quality

The performance of optoelectronic devices strongly depends on the quality of the deposited perovskite layer. Among the factors influencing film morphology, the properties of the underlying substrate play a crucial role in determining the uniformity and smoothness of the final film [27, 40, 41]. In CVD, both the inclination and surface roughness of the substrate significantly impact the reactive sticking probability of the precursors. Specifically, substrates placed at low inclination angles and featuring rough surfaces tend to exhibit a high sticking probability, leading to faster growth but rougher, less uniform films. Conversely, achieving smooth, uniform perovskite layers requires carefully controlled conditions that minimize the reactive sticking probability during growth [42, 43]. To investigate these effects, CsPbBr_3_ layers were grown on both soda‐lime glass (as described in the previous section) and fused silica substrates; for the inclination angle of the substrates, 30° was chosen. Representative SEM images of the corresponding films are shown in Figure 1D,H. Under identical CVD conditions, the CsPbBr_3_ film deposited on fused silica exhibits a markedly improved surface morphology. The resulting layer is smoother, pinhole‐free, and composed of larger grains up to 35 µm in size. The median grain size of the film grown on fused silica (19 ± 0.52 µm, Figure S3) is significantly larger than that of the film deposited on soda‐lime glass (13 µm) (Figure 1C). This difference can be attributed to the distinct surface chemistry and wettability of the substrates [27, 44].

Fused silica consists almost entirely of SiO_2_ (>99.5%), is chemically inert, and presents a smooth (0.6 nm), hydrophilic surface, all of which promote homogeneous perovskite growth. In contrast, soda lime glass contains only about 75% SiO_2_ and additional components such as Na, Ca, Al, K, and SO_2_, which introduce surface roughness (1.5 nm) and chemical heterogeneity [45]. These properties hinder uniform nucleation and result in smaller grain growth. Atomic force microscopy (AFM) measurements further confirm that films deposited on fused silica exhibit slightly lower surface roughness than those on soda‐lime glass (Rq 129.9 ± 16.6 nm vs. 164.5 ± 42.2 nm; Figure 1E,F). The film thickness remains consistent across both substrates, varying only slightly between 2.9 and 3 µm (Figure 1G,I).

The crystal structure of the CVD‐grown CsPbBr_3_ films was analyzed using XRD and Grazing Incidence Wide‐Angle X‐ray Scattering (GIWAXS), as shown in Figure 2A–C. The primary diffraction peaks at 15.2°, 21.6°, 30.8°, and 35.6°, together with weaker reflections between 21.6° and 30.8°, can be indexed to the orthorhombic CsPbBr_3_ perovskite phase The main diffraction peaks correspond in standard Pnma setting to the (101)/(020), (121)/(200)/(002), (202)/(040), and (141)/(301)/(222)/(103) reflections, respectively. Alternatively Pbnm notation can be representing the same space group expressed in a different axes setting [37, 38]. Furthermore, GIWAXS data confirms the polycrystalline nature of the CVD‐grown film consisting of large grains without any prominent directional ordering (Figure 2B).

**FIGURE 2:**
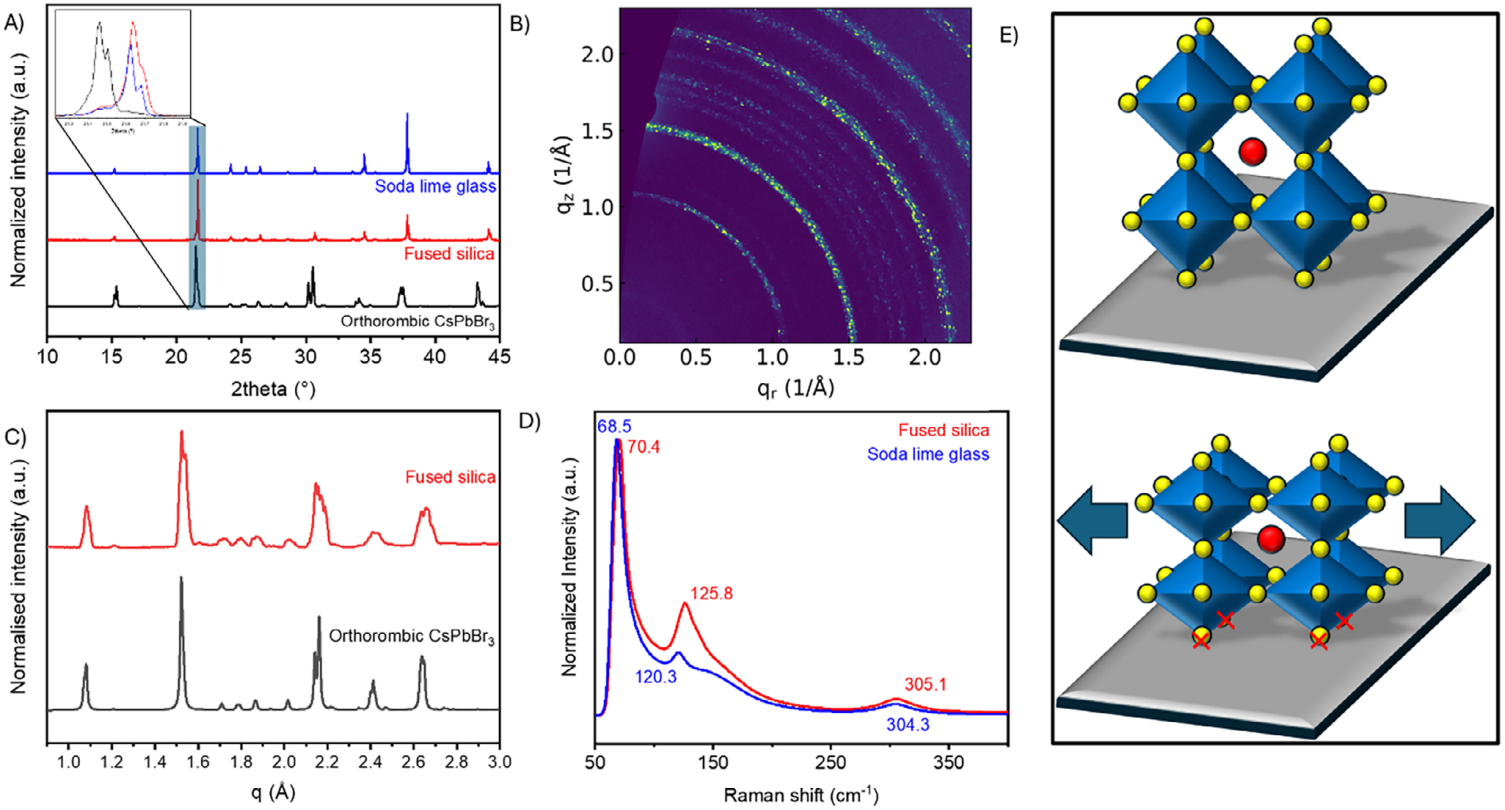
Film crystallinity. (A) Comparison of the XRD patterns between the CsPbBr_3_ samples grown on fused silica and soda lime glass. (B) 2D GIWAXS patterns of CsPbBr_3_ grown on fused silica. (C) Azimuthally integrated GIWAXS pattern of CsPbBr_3_ grown on fused silica. (D) Raman spectra of CsPbBr_3_ grown on fused silica and soda lime glass. (E) Schematic representation of the substrate compressive strain.

Raman spectroscopy, performed with a 633 nm excitation laser (Figure 2D), together with XPS (Figure S6) and EDX mapping (Figures S4 and S5), corroborates these findings. Both the fused silica and glass‐based films exhibit characteristic Raman‐active modes at approximately 70, 125, and 305 cm^−^^1^. The dominant peak at 70 cm^−^^1^ corresponds to the transverse optical (TO_2_) phonon mode, associated with Cs^+^ motion and [PbBr_6_]^4^^−^ octahedral vibrations. The weaker, broader feature at 125 cm^−^^1^ arises from the TO_3_ phonon mode, while the peak near 305 cm^−^^1^ represents a second‐order Raman scattering mode of CsPbBr_3_ [46, 47, 48]. The absence of additional peaks confirms the phase purity of the films and is in good agreement with the XRD results. XPS measurements further confirm the formation of CsPbBr_3_, with the characteristic Cs 3d, Pb 4f, and Br 3d core‐level peaks observed for films deposited on both substrates (Figure S6). No additional core‐level features attributable to secondary phases such as CsPb_2_Br_5_ or unreacted precursors are observed. A systematic shift of the XPS spectra toward higher binding energies (1 eV) is observed for the films grown on fused silica compared to those grown on soda lime glass. Notably, the adventitious C 1s peak exhibits a similar shift, indicating that the displacement affects all measured species equally. This behavior is characteristic of differential surface charging during XPS analysis and does not reflect changes in the chemical composition or bonding environment of the films. The absence of significant changes in peak shape, peak splitting, or relative peak separations further supports the conclusion that both samples consist of the same CsPbBr_3_ phase with a comparable chemical environment. The EDX elemental maps acquired from both plan‐view and cross‐sectional SEM measurements reveal a homogeneous distribution of Cs, Pb, and Br throughout the entire film area and across the full film thickness, indicating uniform composition and consistent film growth. Precise quantification of the Cs:Pb:Br ratio remains inherently limited by the detection sensitivity of EDX in micron‐thick polycrystalline films.

A slight blue shift is observed in the Raman spectra as well as a shift of Bragg reflections in the XRD patterns toward higher diffraction angles for films grown on fused silica compared to those on soda‐lime glass. To evaluate the strain state, an XRD reference pattern of strain‐free CsPbBr_3_ powder was measured. The observed shift toward higher scattering angles indicates lattice contraction, corresponding to compressive stress within the films (schematically illustrated in Figure 2E). The resulting strain originates from the thermal expansion mismatch between the perovskite and the substrate. The thermal expansion coefficients (α) are approximately 4 × 10^−^^5^ K^−^^1^ for CsPbBr_3_, 0.5 × 10^−^^6^ K^−^^1^ for fused silica, and 9 × 10^−^^6^ K^−^^1^ for soda‐lime glass [41, 49, 50, 51]. Upon cooling, the perovskite tends to contract more strongly than the substrate. However, strong film‐substrate adhesion restricts this contraction, inducing lateral tensile strain and compressive strain along the out‐of‐plane direction. This lattice distortion can be accommodated through a slight tilting of the [PbBr_6_]^4^^−^ octahedra, maintaining structural coherence within the perovskite layer [52].

In the fused silica film, the peaks shift to higher diffraction angles because soda‐lime glass exhibits a thermal contraction behavior closer to that of CsPbBr_3_, resulting in reduced intrinsic stress. The observed spectral shift can also be attributed to chemical interactions at the interface between the substrate and the perovskite layer. Interfacial strain in films deposited on soda‐lime glass may arise from alkali‐ion diffusion during high‐temperature deposition, which can interact with the [PbBr_6_]^4−^ octahedral units, weakening the bond strength and reducing phonon vibration frequencies [53, 54]. Cross‐sectional SEM/EDX analysis (Figure S7) of the film‐substrate interface reveals some features that are consistent with this hypothesis. However, the data do not allow a definitive conclusion regarding the extent or nature of interfacial ion diffusion as the expected diffusion would only be trace amounts, and this remains a tentative interpretation. In contrast, fused silica is chemically inert, preserving the lattice structure of the perovskite film. Additionally, XRD analysis reveals peak broadening in films grown on soda‐lime glass, which may be consistent with increased structural disorder at the interface, though contributions from other sources cannot be excluded.″

### 3.3 Impact of Substrate on Film Optical Properties

The optical properties of the CsPbBr_3_ films were investigated using steady‐state photoluminescence (PL) and in situ UV–vis absorption spectroscopy. Figure 3A shows the absorption spectra of the CVD‐grown films, both of which exhibit a distinct absorption onset near 525 nm (2.36 eV). A slight red shift (∼0.02 eV) is observed for the film grown on fused silica. Considering the direct bandgap nature of CsPbBr_3_, the optical bandgaps were estimated from the corresponding Tauc plots (Figure S8) to be 2.32 and 2.34 eV for the films deposited on fused silica and soda‐lime glass, respectively. These values are consistent with previously reported data for bulk CsPbBr_3_ [38]. The disorder in the films was further evaluated via the Urbach energy, extracted from the absorption edge in Figure S8. The CsPbBr_3_ film on fused silica exhibits a lower estimated Urbach energy of 15.39 ± 0.49 meV compared to 17.29 ± 0.45 meV for the film on soda‐lime glass, indicating reduced energetic disorder and fewer defect states in the former.

**FIGURE 3:**
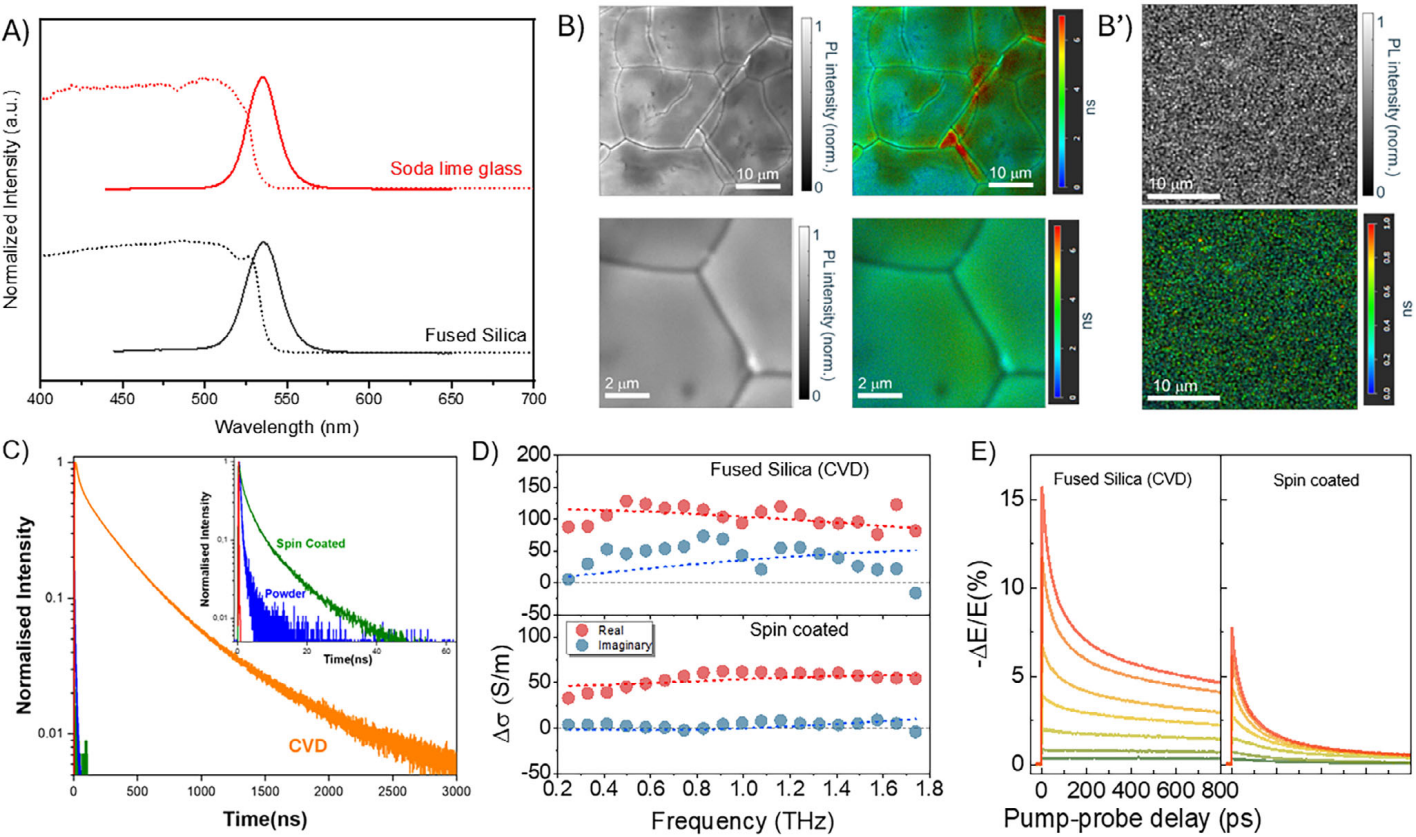
Optical study. (A) UV–vis (dashed lines) and PL (solid lines) spectra of the CVD‐grown films on fused silica and soda lime glass. (B) PL microscopy images of the CVD‐grown film and B’) the spin‐coated film (fused silica). (C) Time‐resolved luminescence decay traces. (D) Complex photoconductivity of CsPbBr_3_ grown via CVD growth and spin‐coating (fused silica), respectively. Measurements 100 ps after the pump. The dashed lines are the fitted results with the Drude/DS model. (E) Fluence‐dependent OPTP dynamics of the spin‐coated and CVD‐grown film (fused silica), with pump fluence from 4.8 to 230 µJ/cm^2^.

The observed bandgap variation can be attributed to substrate‐induced strain. In perovskite materials, the valence band maximum (VBM) is generally more sensitive to lattice strain than the conduction band minimum (CBM) [55]. Under compressive strain, the VBM shifts toward higher energy, effectively narrowing the bandgap. Therefore, the slightly lower bandgap observed for films grown on fused silica is consistent with the higher compressive strain induced by its lower thermal expansion coefficient compared to that of soda‐lime glass [27, 56, 57].

As shown in Figure 3A, the steady‐state PL spectra of both films exhibit a strong green emission. The emission maxima are located at 535 nm (2.32 eV) and 536 nm (2.31 eV) for films deposited on fused silica and soda‐lime glass, respectively, with a full width at half maximum (FWHM) ranging from 0.09 to 0.11 eV. These values align well with previously reported literature data for CsPbBr_3_ thin films [38]. Since both films possess comparable thicknesses, the absence of any notable PL peak shift can be attributed to strain effects rather than optical interference or thickness variation.

### 3.4 Comparison of the Quality of Films Obtained via CVD and Spin Coating

To enable a direct and consistent comparison between CVD‐grown and solution‐processed films, CsPbBr_3_ was also deposited on fused silica substrates via spin coating following a standardized literature protocol [30]. Comprehensive structural and optical characterizations, including SEM, XRD, Raman spectroscopy, profilometry, UV–vis absorption, and photoluminescence (PL) spectroscopy (Figure S9), were performed to confirm phase purity and assess film morphology. The spin‐coated films were significantly thinner (∼200 nm) and exhibited much smaller grain sizes (∼0.3 µm) compared to the CVD‐grown films. These morphological differences are reflected in the steady‐state PL spectra, which display a redshift (∼0.06 eV) in the emission peak for the thicker, CVD‐deposited films (Figure S9). The shift can be attributed to the combination of a larger grain size and increased film thickness, which leads to photon reabsorption [58, 59, 60]. XRD analysis confirms that the spin‐coated sample consists of polycrystalline CsPbBr_3_, with diffraction peaks consistent with the orthorhombic (Pnma) crystal structure reported in the literature [30, 61, 62].

To assess the photogenerated charge carrier dynamics in the films, time‐resolved photoluminescence (TRPL) measurements were performed (Figure 3C). Owing to the slight difference in the emission maxima of the two samples, detection wavelengths at their respective maxima were used, while the excitation wavelength was fixed at 355 nm for both. The PL decay of the spin‐coated sample is completed within 40 ns, whereas the decay in the CVD‐grown sample extends up to 3000 ns.

Considering the large particle size and the low exciton binding energy of CsPbBr_3_, most, if not all, photogenerated carriers are expected to exist as free carriers [63]. Consequently, the prolonged PL decay observed in the CVD‐grown sample indicates that these free carriers can diffuse over longer distances through the material without being significantly impeded by grain boundaries or defects before recombining radiatively [64]. In contrast, the shorter decay time in the spin‐coated films arises from smaller grain sizes, a higher density of grain boundaries, and enhanced non‐radiative recombination due to an increased density of trap states.

These bulk semiconductor systems, which are rich in free carriers [65], typically exhibit non‐exponential recombination kinetics. Accurate modeling of such behavior requires consideration of carrier diffusion, bimolecular recombination, and trap‐assisted recombination processes [66, 67] to extract physically meaningful parameters, an analysis beyond the scope of the present work. Nevertheless, for comparison with previously published reports, in which multiexponential fitting is often applied to such non‐exponential decays, the PL decays were fitted using a triexponential function, and the corresponding time constants are provided in the Supporting Information (Table S2 and Figure S10).

Given the complexity of the PL decay behavior, photon arrival‐time imaging under pulsed excitation was performed to probe the spatial recombination heterogeneity of the films (Figure 3B). Notably, the CVD‐grown film exhibits excellent homogeneity in PL decay across the imaged area. Minor heterogeneity is observed only when the photon arrival time is restricted to the first 7 ns following excitation, where occasional smaller grains display longer photon arrival times within this early‐time window. Although some degree of PL intensity heterogeneity is present, it does not show a clear correlation with photon arrival time and is likely attributable to surface roughness.

In contrast, the significantly smaller grain size and grain‐to‐grain variations in both PL intensity and decay time in the spin‐coated film are clearly evident from the corresponding PL microscopy images (Figure 3B’). Overall, these results highlight the superior optoelectronic quality of the CVD‐grown films and underscore the potential advantages of this fabrication approach for high‐performance optoelectronic applications. The observed differences in recombination dynamics are consistent with the substantial difference in grain size between the two films. Grain boundaries in polycrystalline perovskites are known to act as defect‐rich regions that can facilitate carrier trapping and non‐radiative recombination. Consequently, the reduced grain‐boundary density in the CVD‐grown films decreases the probability of carrier trapping events, enabling photogenerated carriers to remain mobile for longer periods before recombination. This interpretation is supported by the significantly prolonged PL decay observed in the CVD‐grown films.

While TRPL measurements provide information on carrier recombination dynamics, they do not directly probe charge transport. Therefore, ultrafast terahertz (THz) spectroscopy was employed to investigate how the microstructural differences between the spin‐coated and CVD‐grown films influence carrier mobility and localization on ultrafast timescales. In these optical pump–THz probe (OPTP) measurements, the perovskite films were excited by ∼50 fs laser pulses at 400 nm. A subsequent single‐cycle THz pulse was transmitted through the sample, which probes the photoinduced changes in conductivity as Δσ∼e·n·μ, quantified via the relative change in the transmitted electric field (Δ*E*/*E*_0_, where Δ*E*  = *E_pump_*  −  *E*_0_). Here, *e* denotes the elementary charge, *n* the charge carrier density, and *µ* represents the charge carrier mobility. To gain deeper insight into charge transport properties across different crystalline domains, we measured the terahertz time‐domain spectroscopy (THz‐TDS) at the pump‐probe delay time of 100 ps, from which the complex THz photoconductivity in the frequency domain can be inferred:

$$\Delta \sigma \;\left(\omega \right)=-\frac{n_{1}+n_{2}}{Z_{0}l}\times \left(\frac{\Delta E\left(\omega \right)}{E_{0}\left(\omega \right)}\right)$$

where *Z*_0_  =  377 Ω is the impedance of free space, *n*_1_ and *n*_2_ are the refractive indices of the media on both sides of the CsPbBr_3_ film, and *l* is the film thickness, respectively. The experimental Δσ(ω) exhibits a large real component, indicative of free carrier transport, as described by the Drude‐Smith (DS) model, which describes spatially confined charge transport: [38]

$$\Delta \sigma \;\left(\omega \right)=\frac{\omega_{p}^{2}\varepsilon_{0}\tau }{1-i\omega \tau }\;\left(1+\frac{c}{1-i\omega \tau }\right)$$

where ε_0_, τ, and ω_*p*_ represent the vacuum permittivity, carrier scattering time, and plasma frequency, respectively. *c* represents the backscattering probability ranging from 0 (isotropic scattering) to −1 (completely backscattering), providing preliminary evidence of the cross‐boundary transport behavior of free carriers in CsPbBr_3_. In the spin‑coated film, *c* = −0.58, whereas the CVD‑grown film shows a value close to zero (Figure 3D), indicating that charge transport in the CVD‑grown CsPbBr_3_ is well captured by a simple Drude response, without the signatures of charge‑carrier localization associated with the negative c parameter observed for the spin‑coated films, testifying to the superior charge transport properties in CVD samples. The Drude–Smith parameter provides direct insight into the influence of grain boundaries on carrier transport. The strongly negative c value observed for the spin‐coated film indicates substantial carrier backscattering and localization, which are commonly associated with transport barriers arising from grain boundaries and structural disorder. In contrast, the near‐zero c value obtained for the CVD‐grown film indicates that charge carriers experience significantly less backscattering and can move more freely through the polycrystalline network. These results provide direct spectroscopic evidence that the larger grains obtained through CVD growth reduce grain‐boundary‐induced transport limitations. The deviations from a pure Drude response at ∼0.8 and ∼1.8 THz are consistent with polar phonon resonances in CsPbBr_3_, indicating the presence of electron–phonon interactions [68]. The Drude/DS model fitting to the complex Δσ(ω) gives rise to the extraction of carrier density $n=\frac{\omega_{p}^{2}m^{*}\varepsilon_{0}}{e^{2}}\;$ and mobility $\mu =\frac{e\tau (1+c)}{m^{*}}$. Interestingly, the effective scattering time τ(1 + *c*) in these two films are 25 and 54 fs, in the spin coated and CVD grown films, respectively. Using m* = 0.26 *m*_0_ from a previous report [69], the corresponding DC‐limit mobilities are estimated to be 171 ± 23 cm^2^ V^−1^ s^−1^ and 365 ± 54 cm^2^ V^−1^ s^−1^, which accounted for the averaged amplitude change of Δσ(ω),  with nearly the same calculated carrier density. The nearly twofold increase in mobility demonstrates that the microstructural improvements achieved through CVD growth have a measurable impact on carrier transport. Since both films exhibit similar photogenerated carrier densities, the enhanced mobility can primarily be attributed to reduced carrier scattering and localization. Combined with the Drude–Smith analysis, these results indicate that the larger grain size and reduced grain‐boundary density facilitate more efficient charge transport across the film. By varying the delay time between the optical pump and the THz probe pulses, the time‐dependent real part of the photoconductivity is obtained, as shown in Figure 3E. The CVD‐grown film exhibits a doubled amplitude than the spin coated one at all excitation fluences, which is perfectly in line with the doubled carrier mobility inferred from the complex Δσ(ω). In addition, the reduced grain boundary density in the larger crystals promotes radiative recombination, resulting in significantly enhanced charge carrier lifetimes. The observation of a Drude‐like THz response for the CVD sample provides a microscopic link between charge transport and macroscopic device performance, consistent with fast, free‐carrier transport enabled by low trap densities and good crystallinity, which reduce recombination and noise and support efficient, fast, and low‐noise photodetectors. Rigorous determination of the µτ product would require dedicated charge‐collection measurements and is beyond the scope of the present study. However, the combination of prolonged carrier lifetimes observed by TRPL and enhanced carrier mobilities extracted from THz spectroscopy provides strong evidence for improved carrier transport lengths in the CVD‐grown films.

### 3.5 Optoelectronic Performance of CVD Versus Spin‐Coated CsPbBr_3_ Films

Current voltage (I–V) measurements of CVD‐grown CsPbBr_3_ films deposited on fused silica and soda lime glass substrates reveal a marked suppression of dark current in devices fabricated on fused silica (Figure 4A). At 0 V, the measured dark current density falls below the practical detection limit of the Keithley 2400 SourceMeter (>10 pA) and reaches an apparent value of 7.4 × 10^−^^1^ ^3^ A/cm^2^; at 10 V, the dark current remains low at 7.5 × 10^−^^10^ A/cm^2^. This reduction again underscores the superior film quality achieved on fused silica. As charge carrier traps associated with structural defects contribute to leakage currents, the improved crystallinity and morphology naturally result in lower dark current levels. This enhancement is reflected in the switching ratio (I_on_/I_off_), which reaches ∼9 × 10^4^ for devices on fused silica, compared to only ∼2 × 10^2^ for those on soda lime glass, when measured at 10 V. To assess the reproducibility of the electrical characteristics, current–voltage (I–V) measurements were performed on five independently fabricated devices for each substrate type (Figure S11). The devices exhibited highly consistent behavior within each substrate group, indicating good fabrication reproducibility. Furthermore, the observed differences between devices fabricated on fused silica and soda‐lime glass were reproducible across all measured samples, confirming that the substrate‐dependent trends discussed in this work are representative and not the result of device‐to‐device variation.

**FIGURE 4:**
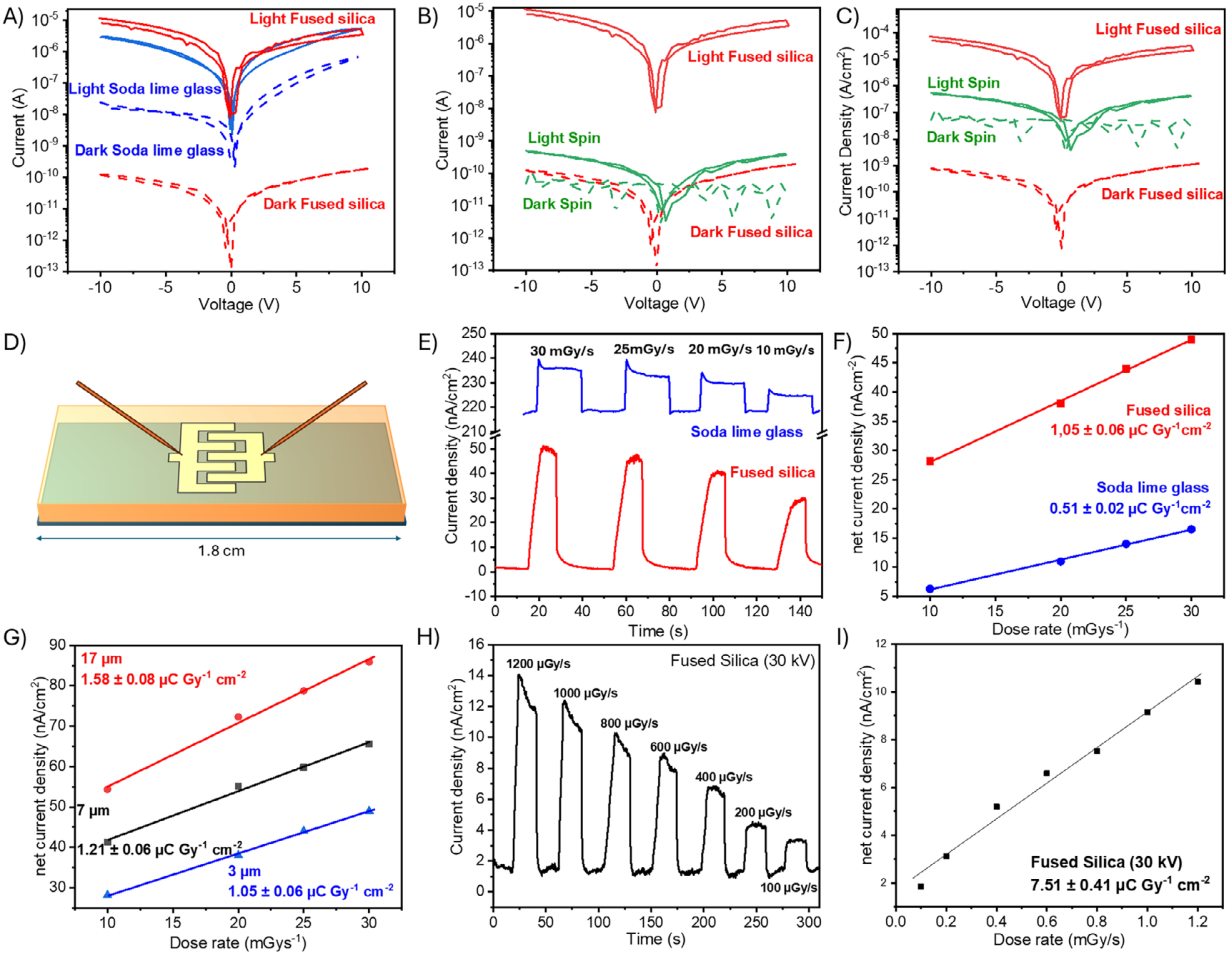
Photodetector device analysis. (A) Current–voltage (I–V) curves of CVD‐grown films on fused silica and soda lime glass. (B) Current–voltage (I–V) curves of CVD‐grown and spin‐coated film on fused silica. (C) Current density–voltage (J–V) curves of CVD‐grown and spin‐coated films on fused silica. A–C are measured under 1‐sun light illumination. (D) Schematic representation of the device with the gold contacts. (E) Current density–time (J–t) curves of CVD‐grown fused silica and soda lime glass films, under X‐ray irradiation (120 kV). (F) Calculated sensitivities for CsPbBr_3_ grown on fused silica and soda lime glass under a 10 V bias. (G) Calculated sensitivities for CsPbBr_3_ grown films on fused silica under a 10 V bias with different thicknesses. (H) Current density–time (J–t) curves of a CVD‐grown fused silica film, under lower X‐ray irradiation (30 kV). (I) Calculated sensitivities for CsPbBr_3_ grown on fused silica and soda lime glass under a 10 V bias.

For spin‐coated devices, current density–voltage (J–V) analysis was used to account for differences in effective device area. A smaller interdigitated mask (40 µm spacing, active area of 1mm^2^) was required to detect a measurable signal due to the lower photocurrent output. Figure 4B shows the corresponding raw current–voltage response for the same two samples; because the CVD‐grown and spin‐coated devices employ electrodes of different active areas (9.5 mm^2^ and 1 mm^2^, respectively), absolute current values in Figure 4B are not directly comparable. As shown in Figure 4C, the CVD‐grown films exhibit both increased photocurrent and significantly suppressed dark current, leading to a substantial improvement in the switching ratio relative to spin‐coated counterparts. To isolate the impact of the deposition method on material quality and device performance, both the CVD‐grown and spin‐coated films were fabricated and evaluated using the same proof‐of‐concept lateral device architecture and identical measurement conditions, rather than optimized vertical device stacks incorporating interface engineering. For context, Table S3 also includes several optimized spin‐coated CsPbBr_3_ photodetectors reported by other groups, with detectivities comparable to or exceeding our own CVD‐grown devices.

Figure 4D depicts a schematic representation of the proof‐of‐concept lateral photodetector device architecture used for the measurements above, in which interdigitated gold contacts (50 nm thickness, 500 µm spacing, 9.5 mm^2^ area) are thermally evaporated onto the semiconductor layer through a shadow mask. This lateral geometry is intentionally employed to probe in‐plane charge transport and noise characteristics intrinsic to the perovskite film, while minimizing the influence of additional charge‐transport layers and interface energetics that dominate vertically stacked device architectures. A xenon arc lamp (100 mW cm^−^^2^) was utilized for sample illumination, and current‐voltage (I–V) characteristics were recorded under both light and dark conditions (Figure 4A–C).

The photodetector performance of the CVD‐fabricated device (fused silica) was evaluated by means of the responsivity (*R*) and specific detectivity (D*):

$$R=\frac{I_{light}-I_{dark}}{P*A}$$

$$D^{*}=\frac{R}{{\left(\frac{2qI_{dark}}{A}\right)}^{1/2}}$$

Here, 𝐼_light_ refers to the photocurrent under illumination, 𝐼_dark_ is the dark current, 𝑃 is the incident light intensity, 𝐴 is the effective irradiated area, and 𝑞 is the elementary charge. Under 1‐sun illumination (100 mW cm^−^^2^), the device exhibits a responsivity of 1.28 × 10^−3^ A W^−^^1^ and a specific detectivity of 6.11 × 10^10^ Jones, both exceeding values reported for comparable devices [70]. Notably, the maximum performance is obtained at lower illumination powers: at 40 µW cm^−^^2^, the responsivity increases to 0.176 A W^−^^1^, and the detectivity reaches 8.40 × 10^12^ Jones. These enhanced values at reduced light intensity are expected, as both responsivity and detectivity typically decline with increasing illumination intensity due to increased carrier recombination [71]. While Lin et al. reported CVD‐grown CsPbBr_3_ photodetectors fabricated on silicon‐on‐insulator (SOI) substrates that exhibit higher responsivity, their detectivity was lower due to a higher dark current (Table S3). This contrast illustrates that our fused silica substrate approach effectively suppresses leakage and noise, enabling higher detectivity [72]. Figure S12 shows the relationship between R, D*, and the power density. The low dark current observed in the CVD‐grown devices is consistent with the transport characteristics revealed by the THz measurements. Reduced grain‐boundary density decreases the number of electrically active interfaces that can facilitate trap‐assisted conduction and carrier recombination, thereby suppressing leakage currents under bias. This interpretation is supported by the larger grain sizes, longer carrier lifetimes, and higher carrier mobility measured for the CVD‐grown films. Grain boundaries play a central role in determining charge‐carrier transport in polycrystalline perovskite films. These interfaces can act as recombination centers, trap sites, and preferential pathways for ionic migration, all of which negatively affect detector performance. Increasing the grain size reduces the total grain‐boundary area per unit volume, thereby lowering the probability of carrier trapping and scattering events. The observed improvements in carrier lifetime, mobility, dark current, and detectivity are therefore consistent with a reduction in grain‐boundary‐mediated losses resulting from the optimized CVD growth conditions. The low noise characteristics of the device are further corroborated by the noise spectral density measurements (Figure S13), which yield a value of 3.2 × 10^−^^1^ ^3^ A Hz^−^^1^^/^^2^ at 100 Hz, confirming that the suppression of grain‐boundary‐mediated leakage pathways translates directly into reduced electrical noise at the device level. It should be noted that the objective of this work is not to achieve record‐setting photodetector performance. As shown in Table S3, several CVD‐grown CsPbBr_3_ photodetectors report higher responsivity or detectivity than the devices presented here, typically achieved through device‐level engineering such as electrode material selection or Schottky‐contact design. Rather, the present work establishes the underlying structure–transport–device relationships that govern how CVD growth conditions influence device performance, a distinction made explicit in Table S4, which compares the characterization scope of representative CVD‐grown CsPbBr_3_ studies against the present work.

To further explore the photodetection capabilities of the fabricated devices, their response under X‐ray irradiation was investigated. (Figure 4E,F) A tungsten target X‐ray tube was employed, with dose rates varying from 10 to 30 mGy s^−^^1^, while a bias voltage of 10 V was applied. From the line shape, we can conclude that the overshoot immediately following illumination may be attributed to rapid photoexcited charge carrier recombination with previously trapped charge carriers [6]. The X‐ray sensitivity (S) was calculated as:

$$S=\left(I_{ph}-I_{d}\right)/\left(D*A\right)$$

where I_ph_ represents the photocurrent, I_d_ the dark current, D the X‐ray dose rate, and A the active area of the device. The sensitivity of devices on fused silica and soda lime glass substrates (1.05 and 0.51 µC Gy^−^^1^ cm^−^^2^, respectively) is markedly lower than that reported for CsPbBr_3_ single crystals (15 µC Gy^−^^1^ cm^−^^2^) measured with a comparable dose rate [73]. This discrepancy is primarily attributed to the limited thickness (3 µm) of the perovskite layer, which leads to insufficient X‐ray attenuation (14%), thereby restricting efficient charge carrier generation. Consequently, a substantial fraction of the incident X‐rays is not absorbed or converted into an electrical signal, ultimately limiting the device's X‐ray detection efficiency. To address this limitation, a thickness‐dependent study was conducted (Figure 4G and Figure S16), in which films of 4, 7, and 17 µm were subjected to the same dose rates. Increasing the film thickness yielded an improved X‐ray sensitivity of 1.58 ± 0.08 µC Gy^−^^1^ cm^−^^2^. Also, reducing the dose rate to the 100–1200 µGy s^−^^1^ range (Figure 4H,I) resulted in a substantial enhancement in sensitivity, reaching 7.51 µC Gy^−^^1^ cm^−^^2^ for the 3 µm film, highlighting the dependence of detector performance on both absorber thickness and incident dose rate. However, increasing film thickness is accompanied by a rise in dark current, which places a practical upper limit on the thickness optimization and must be carefully balanced against the gains in X‐ray sensitivity. It should be noted that the sensitivity values reported here are inherently constrained by the lateral device geometry employed, in which charge carriers traverse a path defined by the electrode spacing rather than the absorber thickness, a configuration fundamentally distinct from vertical stacked architectures in which transport distances are short and collection efficiency is high. Transitioning to a vertical geometry is therefore expected to yield substantially higher sensitivity without requiring further improvement of the absorber material itself, and represents the primary direction for follow‐up work. Interestingly, the photoresponse showed a comparatively weaker dependence on thickness, with the 7 and 17 µm films exhibiting near‐identical photoresponse characteristics, suggesting that beyond a critical thickness the optical absorption is largely saturated and that the improvements observed in X‐ray sensitivity are driven primarily by enhanced X‐ray attenuation rather than changes in the photoactive layer behavior.

To evaluate the stability of the switching behavior in CVD‐grown CsPbBr_3_ films on fused silica, transient photoresponse measurements were conducted using a 405 nm LED as the illumination source. Figure 5A displays the time‐dependent photocurrent (I–t) over repeated on–off light cycles for a total duration of 2500 s (≈42 min). The device exhibits excellent operational stability under periodic switching, indicating reliable, reversible optical switching. Long‐term stability under continuous electrical bias was also assessed. As shown in Figure 5B, devices were subjected to continuous bias (5 V) for over 7 h using a 40 mW lamp. Integrating this setup with the SEM setup also provides visual information on morphological changes during the stability test. The current trace in Figure 5B exhibits two distinct baseline levels, corresponding to the SEM chamber being alternately open (∼10^−^^8^ A, under ambient room light) and closed (∼10^−^^1^ ^1^ A, true dark current), consistent with the ultralow dark currents reported for these devices elsewhere in this work. SEM analysis of the same area before and after irradiation (Figure S14) confirms that no morphological changes or degradation occurred, indicating excellent material and device robustness. This stability is corroborated by the I–t curves, which show that the initial and final switching responses are nearly identical. Figure S15 presents the photo‐response rise time, defined as the time interval between 10% and 90% of the peak photocurrent, which was measured to be 230 ms (Figure S15) and is consistent with values reported for similar planar devices [70, 74]. The decay time (361 ms) exceeds the rise time, as can be seen visually, consistent with the presence of trap states in the material. Upon illumination, photogenerated carriers quickly fill available trap states and reach a saturation regime; when the light is switched off, the delayed release of trapped carriers results in a slower decay of the photocurrent [6].

**FIGURE 5:**
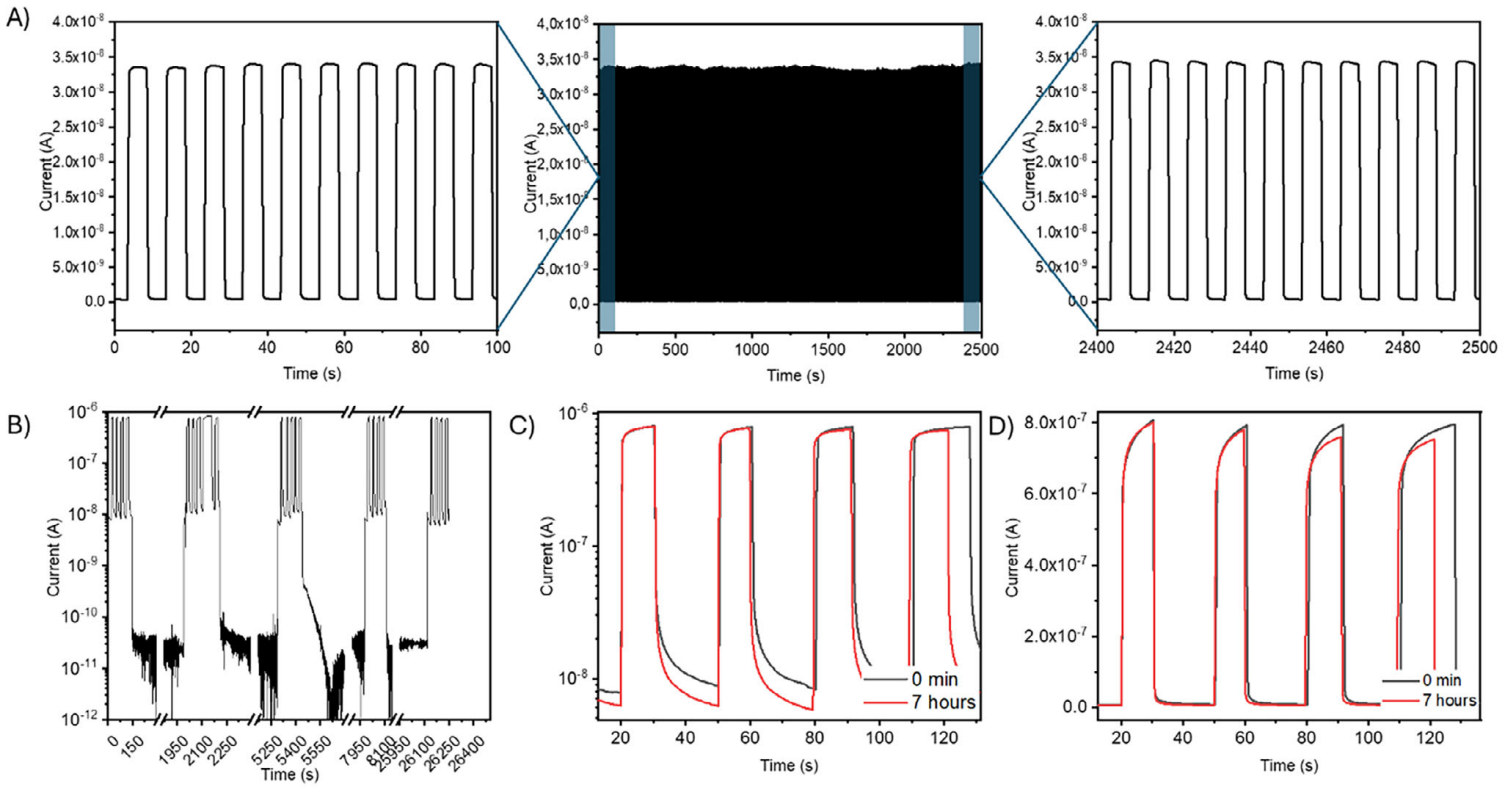
Long‐term stability tests. (A) CVD‐grown film on fused silica, current–time (I–t) curves for 2500 s (≈42 min) under 5 V applied bias and at 40 µW/cm^2^, 405 nm excitation light source. Centre panel = full region, left and right panels = first and last 100 s. (B) Current–time (I–t) trace under continuous electrical bias (5 V) and 40 mW illumination in the integrated SEM setup. The higher current branch (∼10^−^^8^ A) was recorded with the SEM chamber open to ambient light; the lower branch (∼10^−^^1^ ^1^ A) corresponds to the true dark current, recorded with the chamber closed. (C, D) Current–time (I–t) curves to highlight the difference between the first and the last photo‐response measurement (after 7 h), C in log scale and D in linear scale.

To assess the ambient stability of the CVD‐grown CsPbBr_3_ film on fused silica, XRD patterns and long‐term IV stability tests are monitored over a period of one year (Figure S17). Samples were stored on a laboratory shelf under ambient conditions (room temperature, ∼50% relative humidity), without any encapsulation or controlled environment. Remarkably, no discernible changes are observed in the diffraction patterns over the storage period, indicating that the crystal structure of CsPbBr_3_ remains stable under these conditions. These findings highlight the intrinsic structural robustness of CsPbBr_3_ against degradation in air over extended timescales.

Importantly, these results demonstrate that achieving low noise and high detectivity in perovskite photodetectors is not governed by a single parameter, such as grain size or thickness alone, but rather by their coupled influence on carrier transport, recombination dynamics, and defect‐mediated leakage pathways. The present work therefore establishes CVD not merely as an alternative fabrication method, but as a uniquely enabling route for realizing perovskite films that simultaneously satisfy the stringent and often competing requirements of thickness, mobility, noise suppression, and long‐term stability necessary for practical photodetection and X‐ray sensing applications. For this reason, we evaluated the generality of the developed CVD strategy, we extended the process to the lead‐free double perovskite Cs_2_AgBiBr_6_, a chemically more complex multinary system requiring precise stoichiometric control. Using the same reactor geometry, phase‐pure Cs_2_AgBiBr_6_ thin films were reproducibly obtained by appropriate tuning of the precursor configuration and growth parameters. Structural, morphological, and optical characterization confirm high crystalline quality and uniform film formation, comparable to that achieved for CsPbBr_3_, despite the increased chemical complexity. Detailed XRD, SEM, Raman, and optical analyses are provided in the Supporting Information (Figure S18).

## 4 Conclusions

In summary, we demonstrate that chemical vapor deposition enables the fabrication of highly crystalline, large‐grained, and pinhole‐free CsPbBr_3_ films with superior structural and optoelectronic quality compared to their solution‐processed counterparts. By carefully tuning deposition parameters and selecting the substrate, we achieved phase‐pure perovskite films on fused silica, characterized by extended carrier lifetimes and Drude‐like carrier transport. These material advantages translate directly into device‐level improvements, including ultralow dark currents, high responsivity, and detectivities approaching 10^1^ ^3^ Jones, alongside stable and reproducible switching behavior. Furthermore, the intrinsic robustness of CVD‐grown CsPbBr_3_ under ambient conditions underscores its potential for long‐term operational stability. Our findings establish CVD as a reproducible, solvent‐free route to high‐quality halide perovskite films with clear potential for scale‐up and provide a foundation for advancing perovskite‐based photodetectors and X‐ray sensors toward practical applications. Beyond improvements in device performance, this work highlights the relationship between microstructure, carrier transport, and detector operation in CVD‐grown CsPbBr_3_ thick films. By combining grain‐size analysis, photoluminescence spectroscopy, terahertz photoconductivity measurements, and device characterization, we demonstrate that reduced grain‐boundary density leads to enhanced carrier transport and suppressed leakage currents. These findings provide insight into the design of thick perovskite films for future photodetector and X‐ray sensing applications. In particular, transitioning the CVD‐grown CsPbBr_3_ films demonstrated here into vertical‐geometry device architectures is expected to substantially improve X‐ray sensitivity, as the lateral geometry employed in this proof‐of‐concept study inherently limits charge collection efficiency relative to stacked configurations

## 5 Materials Characterization

### 5.1 X‐Ray Diffraction (XRD)

X‐ray diffraction (XRD) patterns of the sample were obtained using a Malvern PANanalytical Empyrean diffractometer, a Cu anode (Cu K_α1_: 1.5406 Å; Cu K_α2_: 1.5444 Å), and a PIXcel3D solid‐state detector. The films were recorded under ambient conditions in a Bragg Brentano geometry with a 2 theta range of 10 to 45 degrees with a step size of 0.0131°. The GIWAXS pattern was recorded at beamline BL9 of the DELTA synchrotron radiation source (Dortmund, Germany) at an angle of incidence of 0.25° with an energy of 13 keV (i.e., a wavelength of 0.9537 Å) and a beam size of 0.05 × 1.0 mm^2^ (vxh). Si powder was used as a calibration sample, and the data were analyzed using pyFAI.

### 5.2 Scanning Electron Microscopy (SEM)

Scanning electron microscopy (SEM) was used to analyze the morphology of the film. The images were obtained by a FEI‐Q FEG250 instrument.

### 5.3 Profilometer

Profilometer was used to analyze the thickness of the films. The profiles were obtained by a DektakXT stylus profiler from Bruker.

### 5.4 Atomic Force Microscopy (AFM)

Atomic force microscopy (AFM) was obtained in ambient conditions on the MultiMode 8‐HR (Bruker) operating in tapping mode with OMCL‐AC160TS R3 (∼26 N/m) probes. Experiments were repeated for three different areas of each sample. The images were processed and analyzed using the software Gwyddion 2.55.

### 5.5 In situ UV–Vis Diffuse Reflectance

In situ UV–vis diffuse reflectance spectra of the films were measured in an integrating sphere of a commercial Perkin‐Elmer Lambda 950. The reflectance was later converted by the Kubelka–Munk theory.

### 5.6 Steady‐State Photoluminescence (PL)

Steady‐state photoluminescence (PL) spectra were measured with an FLS980 fluorimeter. The excitation wavelength was 410 nm, and the emission range was scanned between 440–650 nm.

### 5.7 Photon Arrival‐Time Imaging

Photon arrival‐time imaging was performed using a Leica SP8 confocal microscopy setup (TCS SP8 multiphoton system) with a 100x oil objective (HC PL APO 100x/1.40 OIL) and HyD detectors. Excitation wavelengths were set to 405 nm using a pulsed laser at a 5 MHz repetition rate and 50 nW laser power. Images were acquired using a fast‐scanning module with 512 × 512 pixels (pixel size  =  240 nm). Photons that arrive within a set time window after each pulse were collected. The window is defined by the instrument IRF and the upper limit of the time marked in the color scale of the corresponding image. The image is constructed based on the arrival time of the photons in each pixel after each pulsed excitation.

### 5.8 Terahertz Photoconductivity Measurements

Terahertz photoconductivity measurements were used to probe the photoconductivity of perovskite films with both temporal and spectral resolution. The experiments employ a Ti:sapphire mode‐locked regenerative amplifier delivering ∼50 fs pulses at 1.55 eV with a 1 kHz repetition rate. 3.10 eV excitation is obtained via frequency doubling using a BBO crystal. Single‐cycle THz pulses (∼1 ps duration) are produced and detected using 1 mm (110)‐oriented ZnTe crystals via optical rectification and free‐space electro‐optic sampling, respectively. All measurements are conducted at room temperature in transmission mode under a dry N_2_ atmosphere.

### 5.9 Microsecond Time‐Resolved Luminescence

Microsecond time‐resolved luminescence was used to measure the fluorescence decay times of the CVD‐grown films. A 395 nm laser pulse (8 ns, 10 Hz) generated by a system consisting of a pulsed Nd:YAG laser (Quanta‐Ray INDI‐40, Spectra Physics) was used to excite the samples. The excitation light was focused on the sample by a 150‐mm focal length lens, and a small part of this light was sent to a fast photodiode to generate a trigger signal. A right‐angle configuration between excitation and light collection paths was used, and the luminescence was collected, filtered, and focused on the entrance slit of a 30 cm focal length monochromator. A SpectroPro‐300i monochromator/spectrograph was used to disperse the emitted light, and 395 nm was selected as the excitation wavelength. The optical signal was detected by a PMT (Hamamatsu, R928) and the transient electrical signal was amplified and sent to a computer‐controlled oscilloscope.

### 5.10 Time‐Correlated Photon Counting (TCSPC)

Time‐Correlated Photon Counting (TCSPC) was used to measure the fluorescence decay times at the nanosecond time scale for spin‐coated and Powder samples. The frequency‐doubled output (395 nm, 8.18 MHz, 2 ps FWHM) of a mode‐locked Ti:Sapphire laser (Tsunami, Spectra Physics) was used as the excitation source. The linearly polarized excitation light was rotated to a vertical direction by the use of a Berek compensator (New Focus) in combination with a polarization filter and directed onto the samples. The emission was collected under 90° with respect to the incident light and guided through a polarization filter that was set at the magic angle (54.7°) with respect to the polarization of the excitation beam. The fluorescence was spectrally resolved by a monochromator (Sciencetech 9030, 100 nm focal length, wavelength accuracy 0.3 nm), and detected by a microchannel plate photomultiplier tube (MCP‐PMT, R3809U‐51, Hamamatsu). A time‐correlated single photon timing PC module (SPC 830, Becker & Hickl) was used to obtain the fluorescence decay histogram in 4096 channels. The decays were recorded with 10 000 counts in the peak channel. The data were analyzed with a time‐resolved fluorescence analysis (TRFA) software based on iterative re‐convolution of the data with the instrumental response function (IRF). The full width at half maximum (FWHM) of the IRF was typically in the order of 25 ps.

### 5.11 I‐V, I‐t Photoconductivity Measurements

I‐V, I‐t photoconductivity measurements were perfor devices with a Keithley 2400 SourceMeter. For this measurement Au electrodes (50 nm) needed to be deposited onto the perovskite layer with a 500 µm spacing. To generate 1 Sun (100 mW/ cm^2^), we used an X‐Cite 120 PC Q fluorescence illumination system.

### 5.12 X‐Ray Detection Measurements

X‐ray detection measurements were measured for devices with a CIX2 Radiation Cabinet from Xstrahl life sciences. The X‐ray source can generate X‐rays up to 220 kV. To measure the dose a Romeo dosimeter was employed.

### 5.13 Photo Response Stability Measurements Under Continuous Bias

Photo response stability measurements under continuous bias were conducted in an integrated SEM. Devices were biased at a constant voltage of 5 V while being illuminated with a broadband lamp delivering an optical power of 40 mW. Current–time (I–t) measurements were continuously recorded to monitor the temporal evolution of the photo response.

### 5.14 XPS

XPS measurements were carried out on an Kratos Axis Ultra DLD imaging photoelectron spectrometer. Measurements were performed using the instrument´s Hybrid mode with a monochromated Al K(alpha) source operating at 10 mA emission current and 15 kV voltage bias in a UHV chamber with a base pressure of approx. 1 × 10‐9 mbar. The source line width is approximately 1.0 eV FWHM, as calibrated with the Ag 3d5/2 line. The analysis area was 700 µm × 300 µm. The data were collected using a hemispherical analyzer at an angle of 0° to the surface normal. The takeoff angle was kept fixed along the surface normal in all experiments. Survey spectra were measured at a pass energy of 80 eV and elemental spectra at 20 eV pass energy.

## Author Contributions

R.V.B. conducted most of the experiments and their analysis, and wrote the manuscript under the supervision of E.D. Time‐resolved spectroscopy was performed by D.K. and S.S.; S.S. analyzed the data and wrote the corresponding sections under the supervision of J.H. B.P. measured and analyzed the GIWAXS data together with C.S. A.B. completed atomic force spectroscopy and analysis under the supervision of S.D.F. L.G. performed THz measurements and analysis under the supervision of H.I.W. and M.B. W.Q. assisted in the analysis of the photoconductivity measurements. W.X and F.Z. assisted in the statistical analysis of the photoconditivty. The research line presented in the manuscript was conceived by J.H. and E.D. All authors have contributed to the revision of the manuscript text.

## Conflicts of Interest

The authors declare no conflicts of interest.

## Supporting information




**Supporting File**: advs77049‐sup‐0001‐SuppMat.pdf.

## Data Availability

The data that support the findings of this study are available from the corresponding author upon reasonable request.
