# Supplementary material for: Engineering Large‐Grain Metal Halide Perovskite Films via Chemical Vapor Deposition for High‐Performance, Ultralow‐Noise Perovskite Photodetectors
Source: Adv Sci (Weinh). 2026 Aug 10:e77049. Online ahead of print. doi: 10.1002/advs.77049 (PMC13456116; doi:10.1002/advs.77049)
Supplement: Supplementary file 1 [file ADVS-9999-e77049-s001.pdf]

Supporting information for

Engineering Large-Grain Metal Halide Perovskite films via Chemical Vapor Deposition for High-Performance, Ultralow-Noise Perovskite Photodetectors

Roel Vanden Brande[1], Sudipta Seth[1], Lei Gao[2], Bapi Pradhan[1], Dilkhush Khicher[1], Anastasiia Bazylevska[1], Wei Qu[1], Christian Sternemann[3], Hai Wang[2], Steven De Feyter[1], Weidong Xu[4], Fei Zhang[5], Mischa Bonn[2], Johan Hofkens[1,2] and Elke Debroye*[1]*

[1]Molecular Imaging and Photonics, Department of Chemistry, KU Leuven, Celestijnenlaan 200F, 3001 Leuven, Belgium

[2]Max-Planck-Institute for Polymer Research, 55128 Mainz, Germany

[3]Fakultät Physik/DELTA, Technische Universität Dortmund, Maria-Goeppert-Mayer-Straße 2, 44227 Dortmund, Germany

[4]Northwestern Polytechnical University, 127 West Youyi Road, Xi'an Shaanxi, 710072, China

[5]Henan University, 379 Mingli Road, Zhengzhou, China

**Corresponding emails*: elke.debroye@kuleuven.be

Common failure modes during film preparation.

To improve reproducibility and provide practical guidance for the preparation of high-quality thin films, we summarize common failure modes encountered during processing, their likely physical or chemical origin, and recommended mitigation strategies. These observations are based on repeated experimental trials and were found to be critical for achieving uniform, stable, and reproducible device performance.

Table S1: Reproducibility guidelines for CVD thin-film growth.

Issue observed	Likely cause	Impact on film/device	What to avoid	Recommended mitigation
Non-uniform film thickness along the substrate	Temperature gradient or non-uniform precursor flux	Spatially varying conductivity and photoresponse	Placing substrates outside the isothermal zone	Position substrates in the furnace center; verify temperature profile
Sparse or incomplete surface coverage	Insufficient precursor vapor pressure	Discontinuous films; high leakage currents	Too low source temperature or carrier gas flow	Optimize source temperature and carrier gas flow rate
Parasitic deposition upstream	Premature precursor condensation	Depletion of active species at substrate	Cold spots near precursor zone	Minimize temperature gradients; insulate cold regions
Secondary phase formation	Incorrect precursor ratio; differential evaporation rates; decomposition	Reduced stability; degraded photoresponse	Off-stoichiometric precursor placement; premature evaporation	Precise precursor weighing; separate precursor zones if needed

Uncontrolled nucleation density	Excessive supersaturation at substrate	Rough films; grain-boundary-dominated transport	High precursor flux during early growth	Gradual temperature ramp; delayed substrate exposure
Large grain size dispersion	Unstable supersaturation conditions	Increased device-to-device variability	Rapid temperature ramping	Use controlled heating and cooling ramps
Poor crystallinity	Suboptimal substrate temperature	Low carrier mobility; broadened optical features	Growth at too low temperature	Optimize substrate temperature for surface diffusion
Particle or powder deposition on film	Precursor spattering or condensation upstream	Electrical shorts and surface roughness	Excessively high precursor temperature	Reduce precursor temperature; increase source–substrate distance
Film delamination after growth	Thermal mismatch or rapid cooling	Mechanical and electrical failure	Rapid furnace cool-down	Controlled cooling inside furnace
Reactor contamination between runs	Residual precursor deposition on tube walls	Poor batch-to-batch reproducibility	Skipping tube cleaning	Regular reactor cleaning and conditioning cycles

The precursor powders were pregrinded to initiate a partial reaction prior to the CVD synthesis. Full conversion can be achieved by melting the pregrinded mixture at 550 °C, followed by cooling to room temperature. As shown in the XRD patterns, the pregrind powder already exhibits characteristic reflections of $CsPbBr_3$, whereas the premelted sample yields phase-pure $CsPbBr_3$.

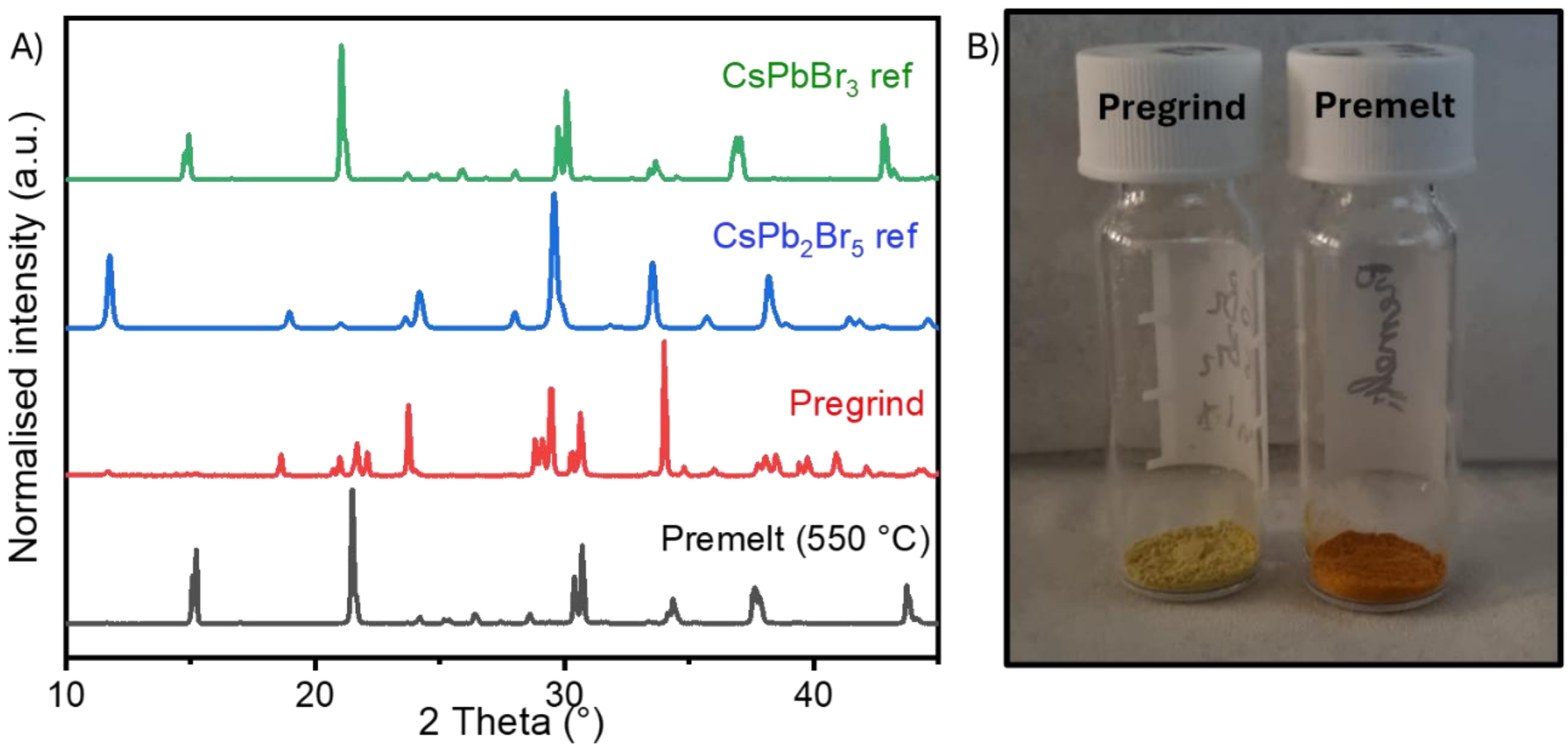

Figure S1. (A) XRD pattern of partially reacted CsBr and $PbBr_2$ after grinding in a mortar and premelting. (B) Yellow powder is the pregrinded material and the orange powder is the premelted material.

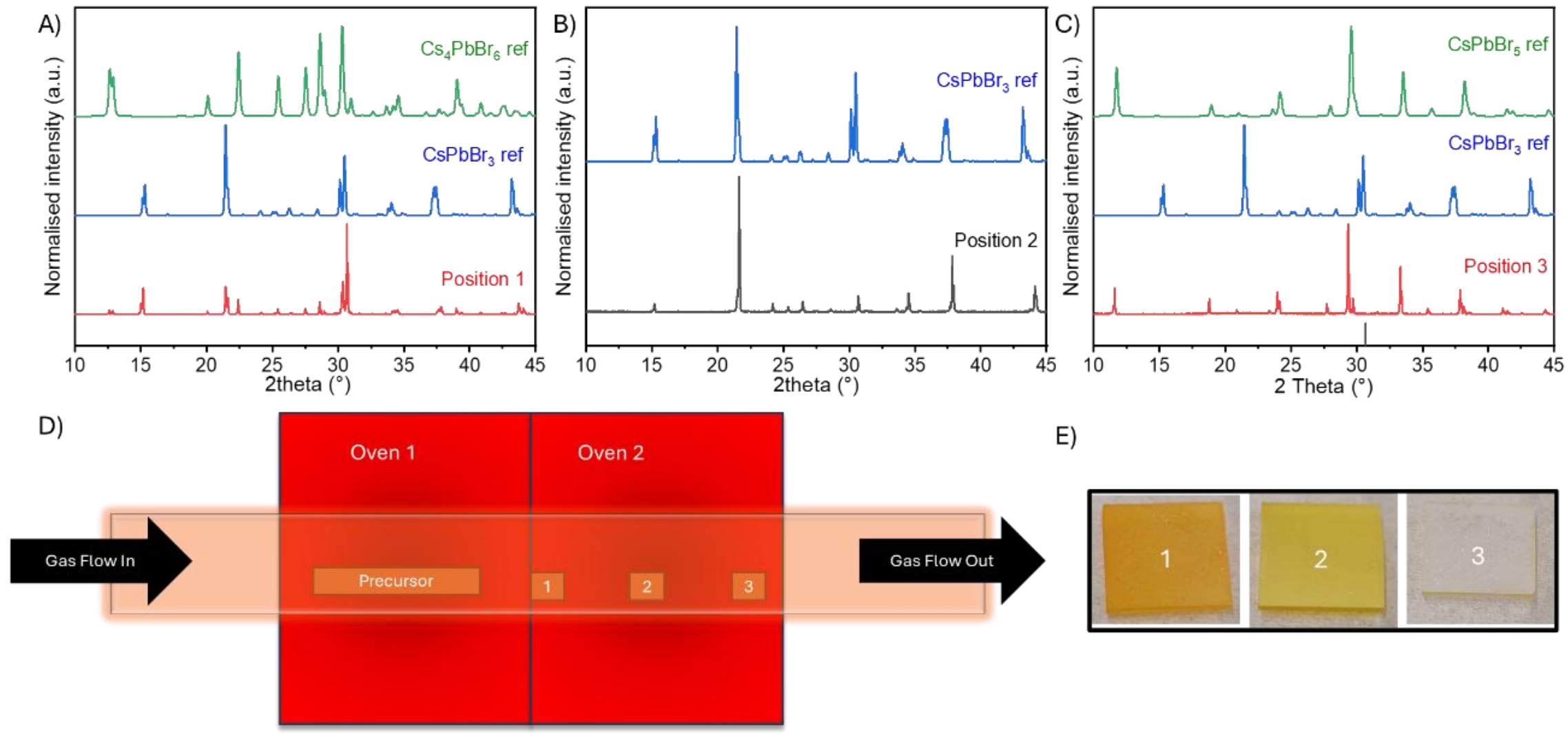

Figure S2. (A-C) XRD patterns of $CsPbBr_3$ films obtained at different substrate positions within the CVD reactor. (A) At the entrance of the second heating zone (closest to oven 1), where the strongest temperature gradient induces a Cs-rich environment, the films contain a Cs_4PbBr_6 secondary phase. (B) At the central position of oven 2, a phase-pure $CsPbBr_3$ film is obtained. (C) At the end of oven 2, due to the increased volatility of $PbBr_2$ the formation of $CsPb_2Br_5$ as a secondary phase is favored. (D) Schematic representation of the CVD setup and substrate positions. (E) Photographs of the corresponding films.

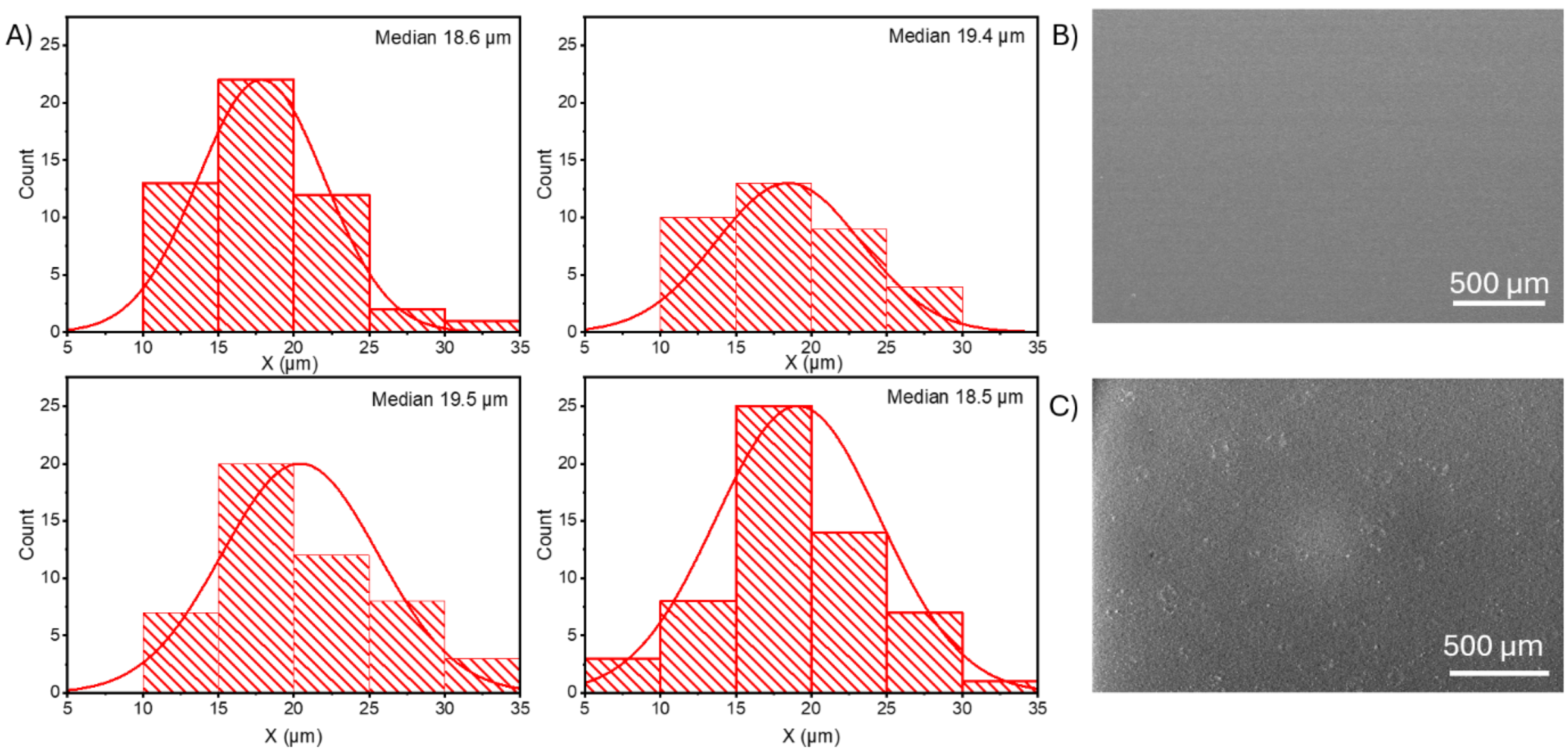

Figure S3.(A) Quantitative grain-size statistics based on SEM analysis of 50 grains per sample. (B) Large-area SEM image of a typical CVD grown film on fused silica and (C) on soda lime glass.

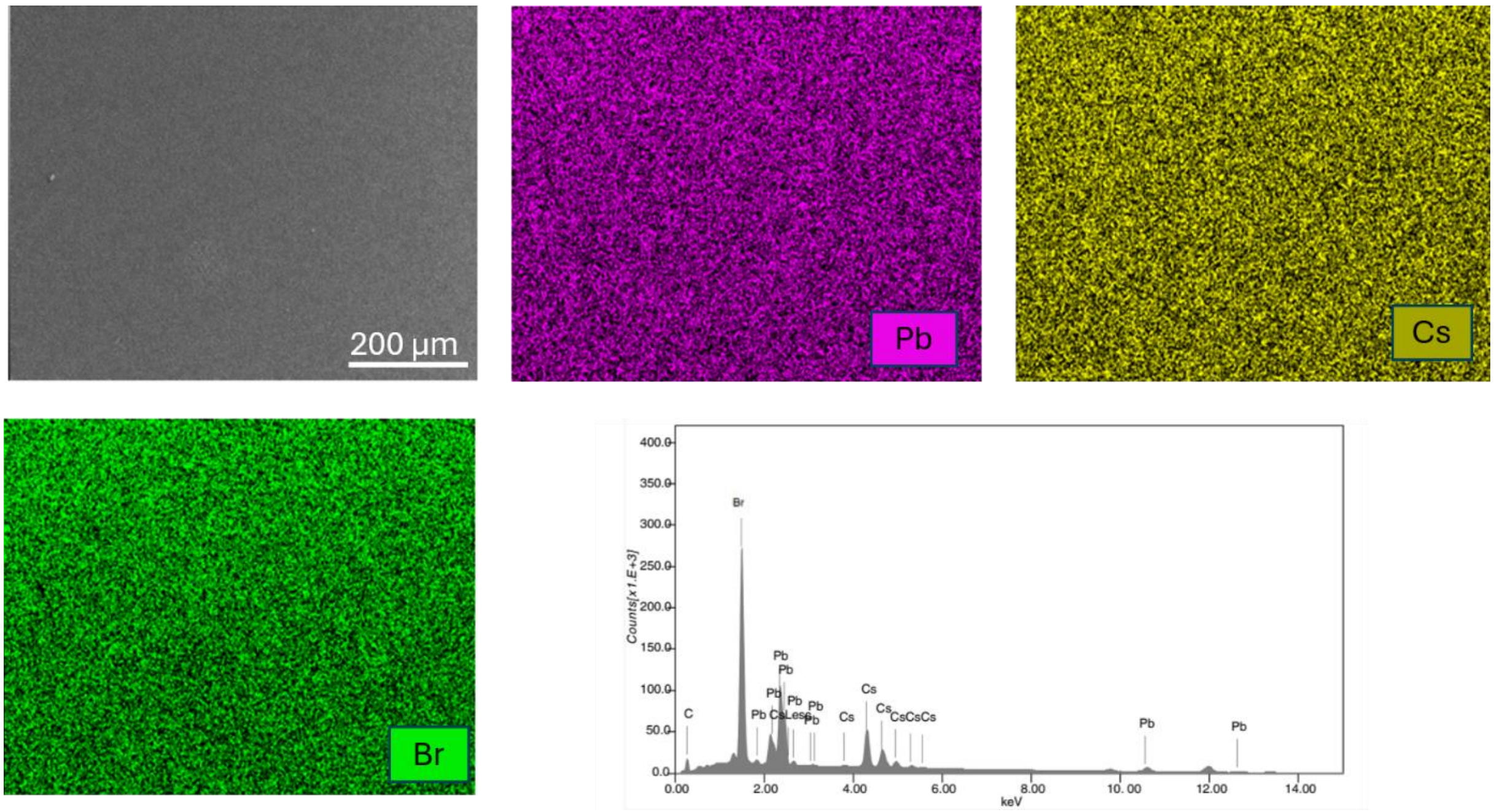

Figure S4. SEM image and corresponding EDX elemental maps of a CVD-grown $CsPbBr_3$ film on fused silica, showing the spatial distribution of Cs, Pb, and Br.

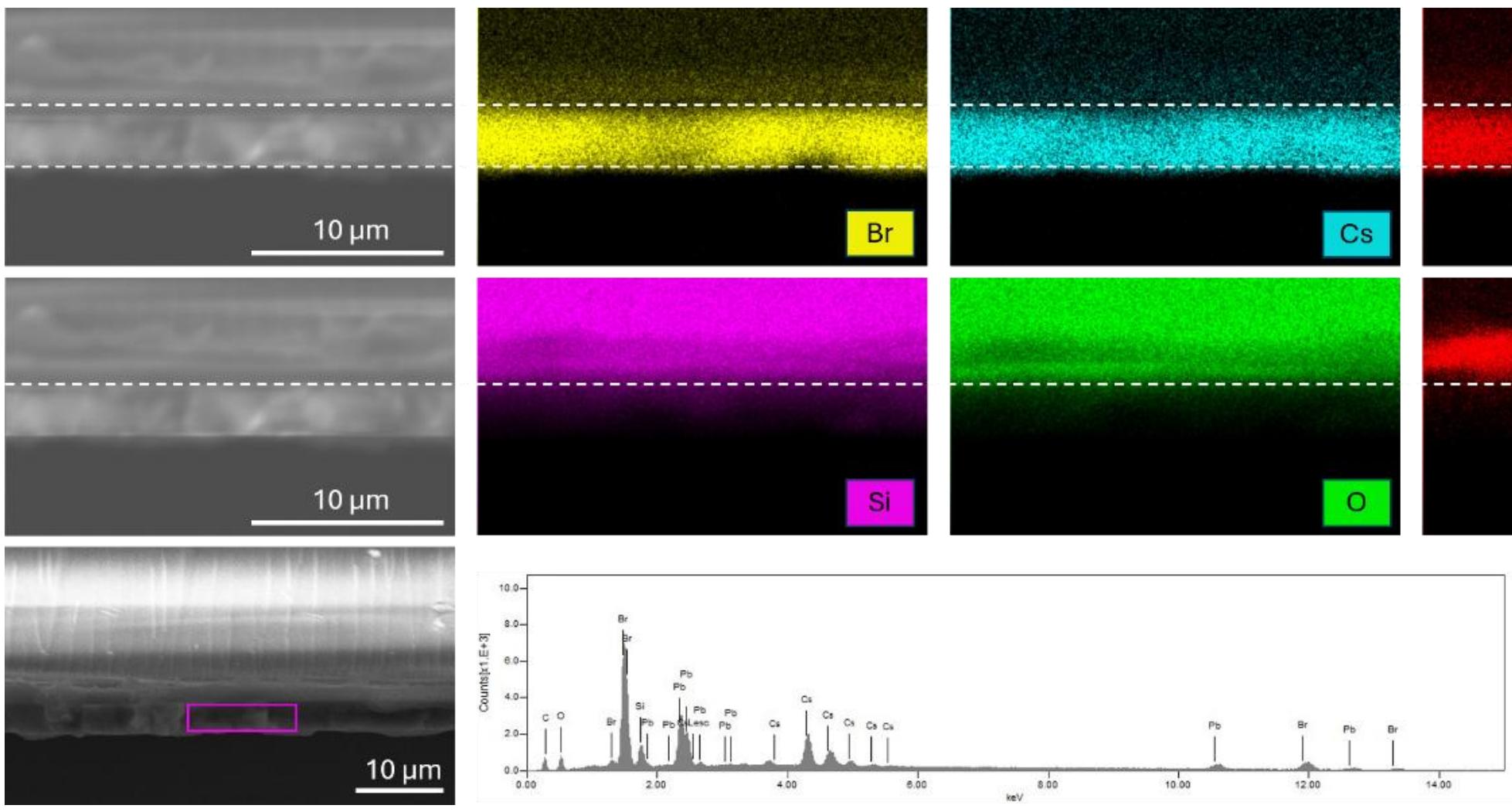

Elements	Mass %	Atom%
Cs	30,87	19,74
Pb	48,96	19,96
Br	19,24	60,03

Figure S5. Cross-sectional SEM image and corresponding EDX elemental maps of a CVD-grown $CsPbBr_3$ film on fused silica, showing the lateral homogeneity and vertical distribution of Cs, Pb, and Br across the film thickness.

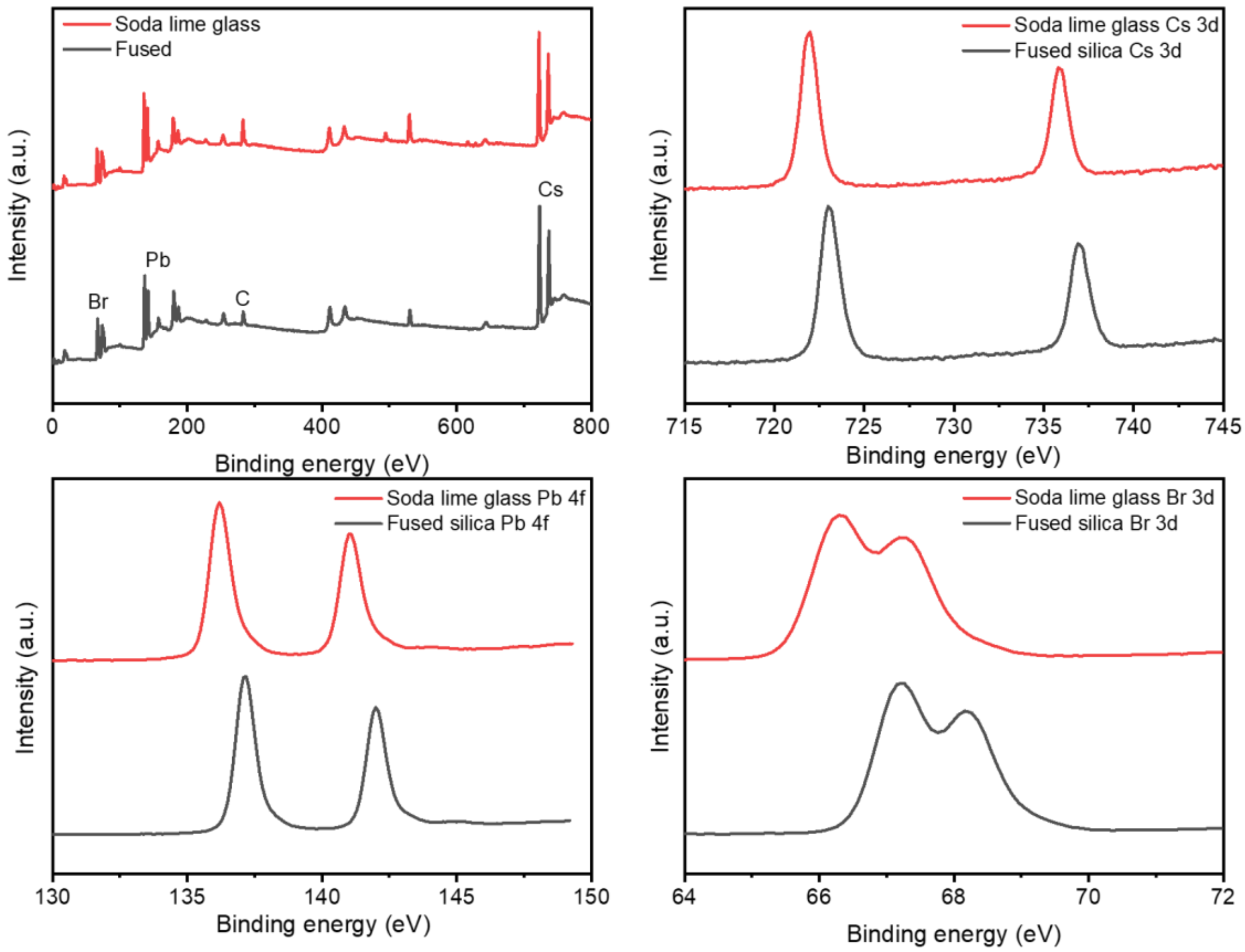

Figure S6. XPS spectra of $CsPbBr_3$ films grown on fused silica and soda-lime glass, showing the Cs 3d, Pb 4f, and Br 3d core-level regions. All spectra are referenced to the adventitious carbon C 1s peak at 284.8 eV.

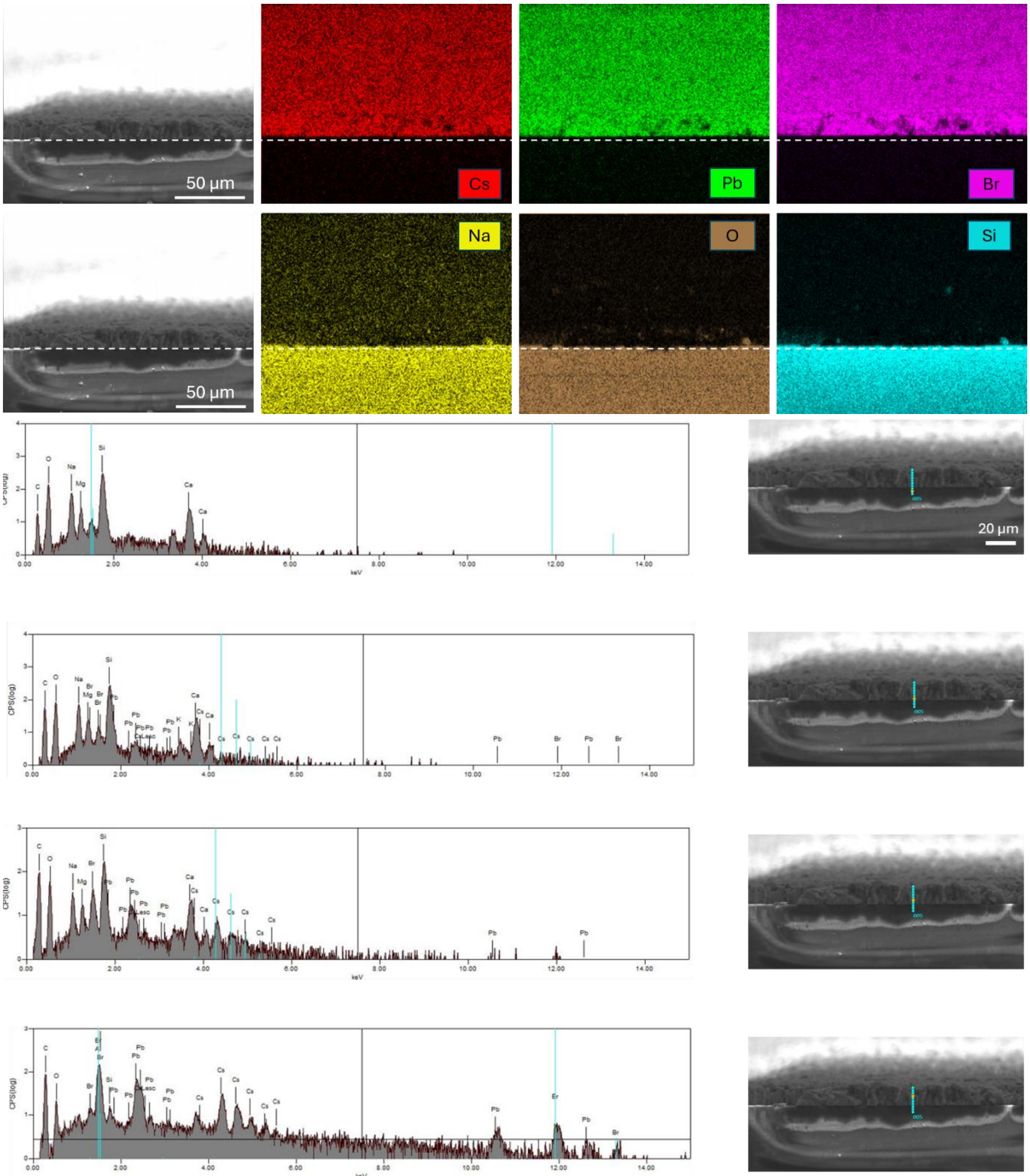

Figure S7. Cross-sectional SEM image and corresponding EDX elemental maps of a CVD-grown $CsPbBr_3$ film on soda-lime glass (~17 µm thick). EDX signals for Na and K within the perovskite layer are suggestive of alkali ion diffusion from the glass substrate into the film.

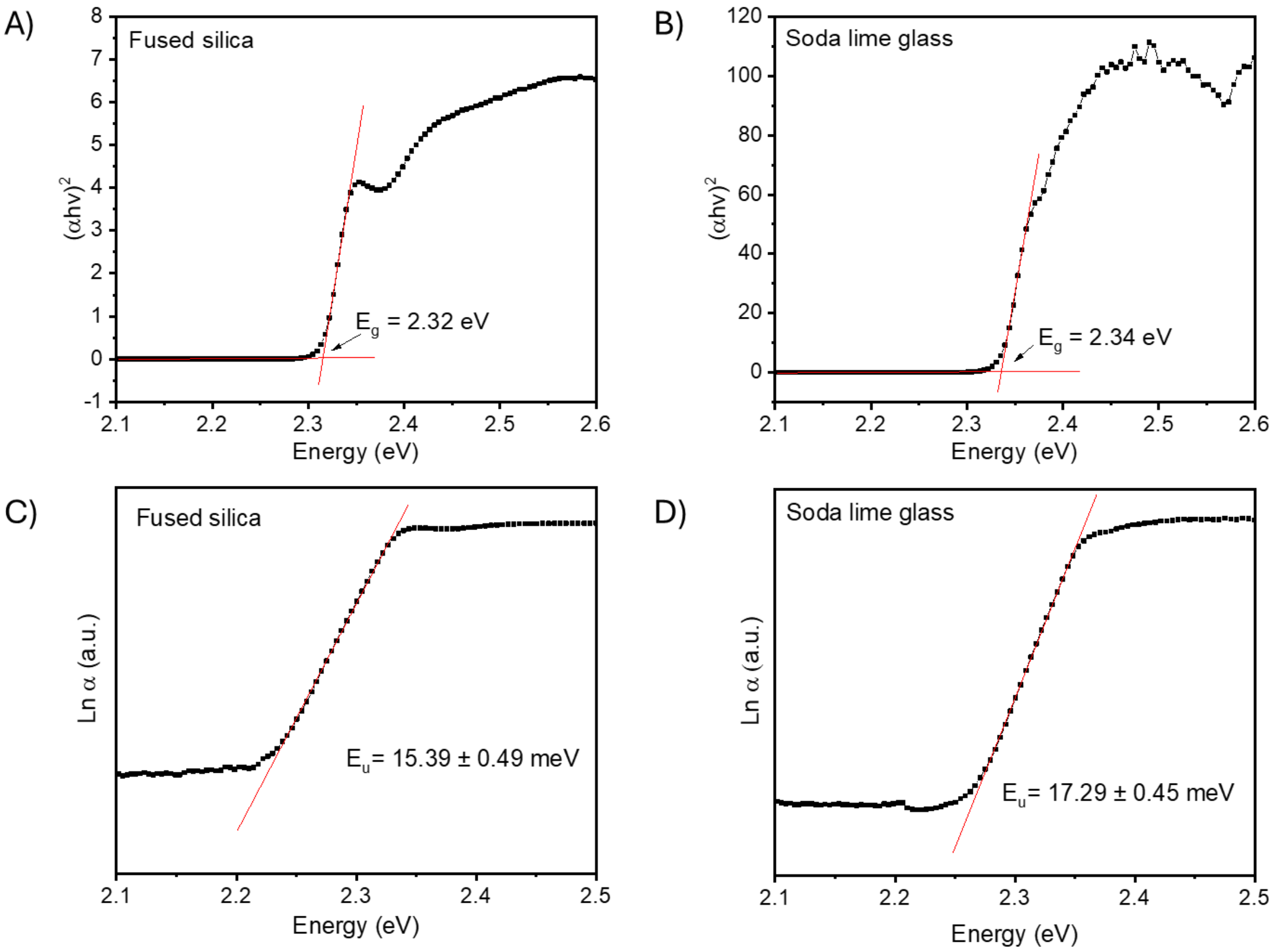

Figure S8. (A-B) Tauc plot analysis for the bandgap estimation of $CsPbBr_3$ grown on (A) fused silica and (B) Soda lime glass.(C-D) Urbach tailing fit and Urbach energy estimation of $CsPbBr_3$ grown on (C) fused silica and (D) Soda lime glass.

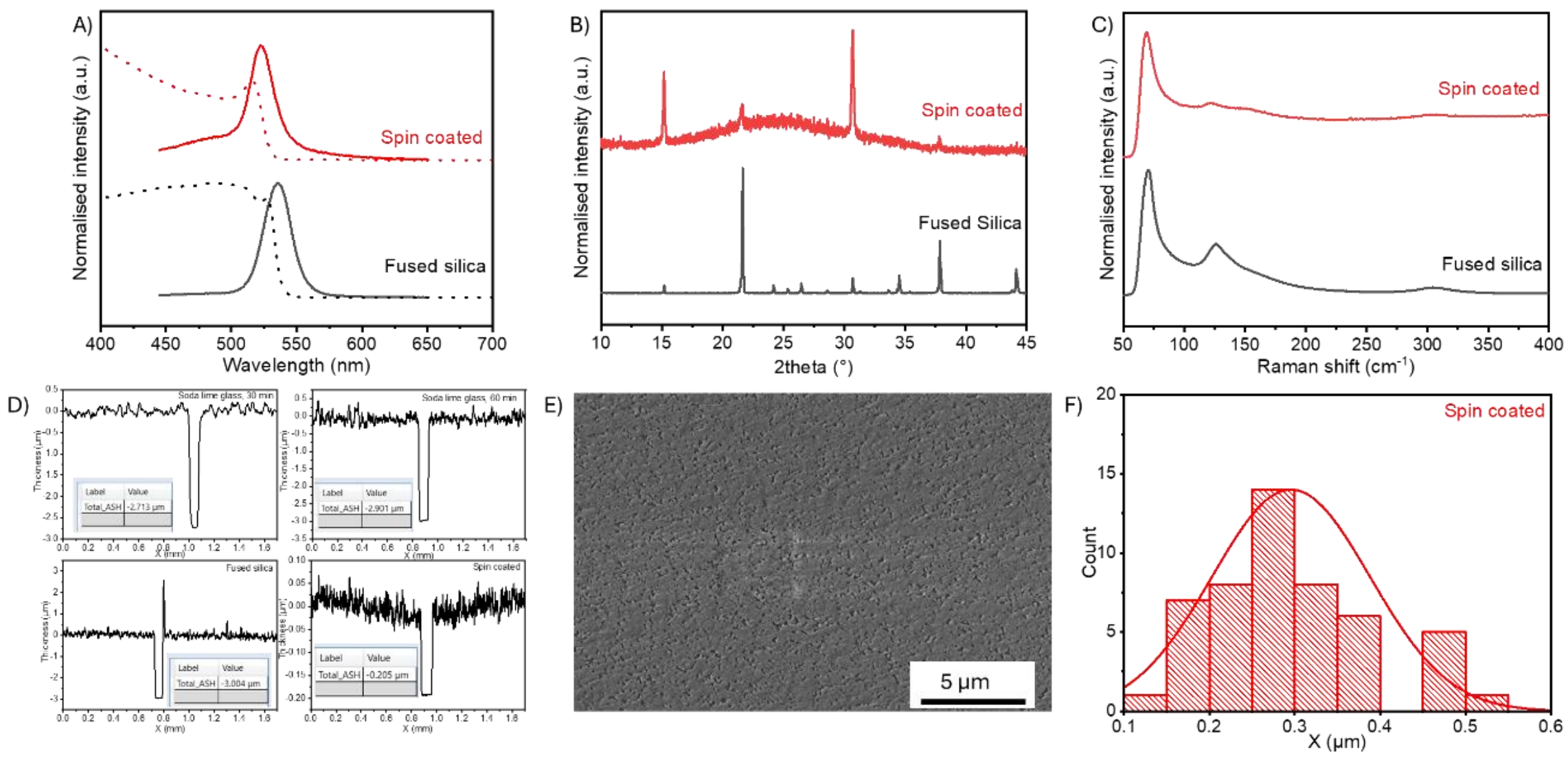

Figure S9. Comparative characterization of $CsPbBr_3$ films prepared by CVD and spin coating. (A) UV–Vis absorption and steady-state PL spectra, showing a redshift of ~0.06 eV in the emission peak for the thicker CVD-grown films relative to the spin-coated films. (B) XRD patterns illustrating the $CsPbBr_3$ phase in spin-coated films, in contrast to the orthorhombic phase obtained via CVD growth. (C) Raman spectra of CVD grown and spin-coated $CsPbBr_3$ films. (D) Film thickness determined by profilometry, revealing significantly thinner spin-coated films (~200 nm) compared to the CVD-grown counterparts. (E) SEM image of the spin-coated film, displaying a fine-grained microstructure, with pinholes. (F) Grain size distribution of the spin-coated film, demonstrating an average grain size of ~0.3 μm.

PL decay curves fitted using a multi-exponential model:

$$I(t) = \sum_{i=1}^{n} A_i \exp(\frac{-t}{\tau_i})$$

where A_i and τ_i denote the amplitude and decay time of the i-th component, respectively. Each PL decay curve can be fitted by a triple exponential function. Here τ_1 can be attributed to relatively fast, defect-mediated recombination, τ_2 can be attributed to exciton-based radiative recombination and τ_3 can be attributed to the recombination of free carriers.[1] A significantly prolonged decay time was observed in the CVD-grown film of ~296 ns compared to the lower excitonic PL lifetime of ~15 ns for the spin coated counterpart. This prolonged lifetime indicated that free charge carriers can diffuse for longer distances throughout the material without being hindered by grain boundaries or other defects. The reduced lifetime in the spin coated films strongly suggests enhanced non-radiative recombination originating from a higher density of trap states. Time-resolved PL imaging (Figure 3B) allowed to map the PL decay times at the grain boundaries. As the relative contribution of grain boundaries increases due to the smaller grains in the spin coated sample, the corresponding trap-assisted recombination is more prominent. These observations highlight the superior optoelectronic quality of the CVD-grown films and underscore the advantages of this fabrication method for high-performance photodetectors.

Table S2. Fitting parameters extracted from time-resolved photoluminescence decay curves of the different samples.

**Samples**	**τ_1 (ns)**	**A_1%**	**τ_2 (ns)**	**A_2%**	**τ_3 (ns)**	**A_3%**	**τ_{avg} (ns)**
CVD	65.6	36.46	346	58.33	1357	5.21	296.4
Spin coated	6.42	35.02	18.79	64.98	/	/	14.5
Powder	0.37	88.68	3.9	11.32	/	/	0.77

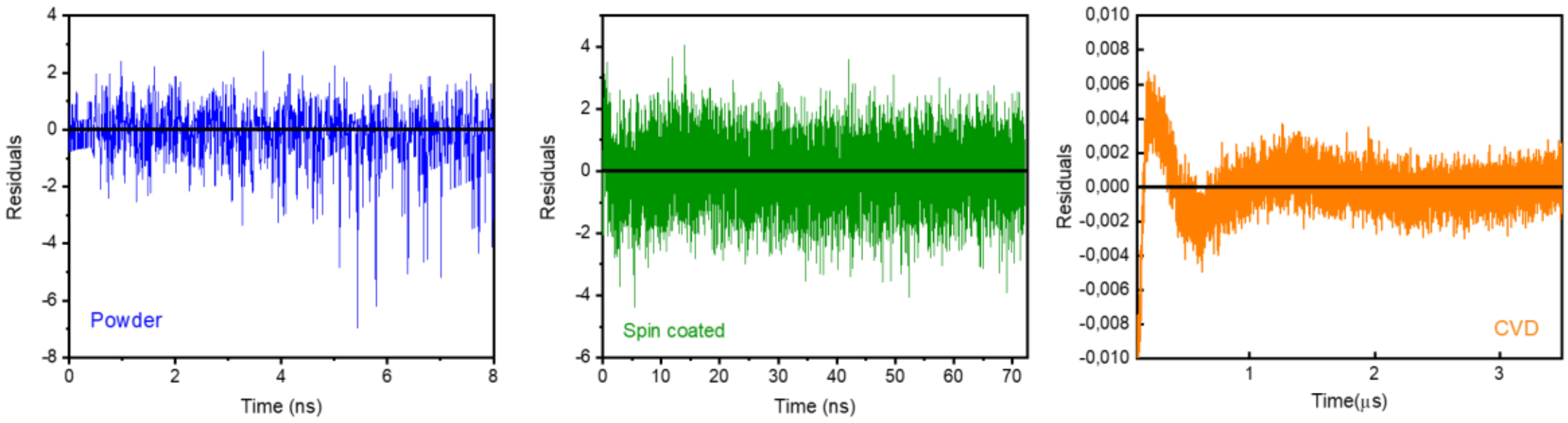

Figure S10. The residual of biexponential fitting of the lifetimes.

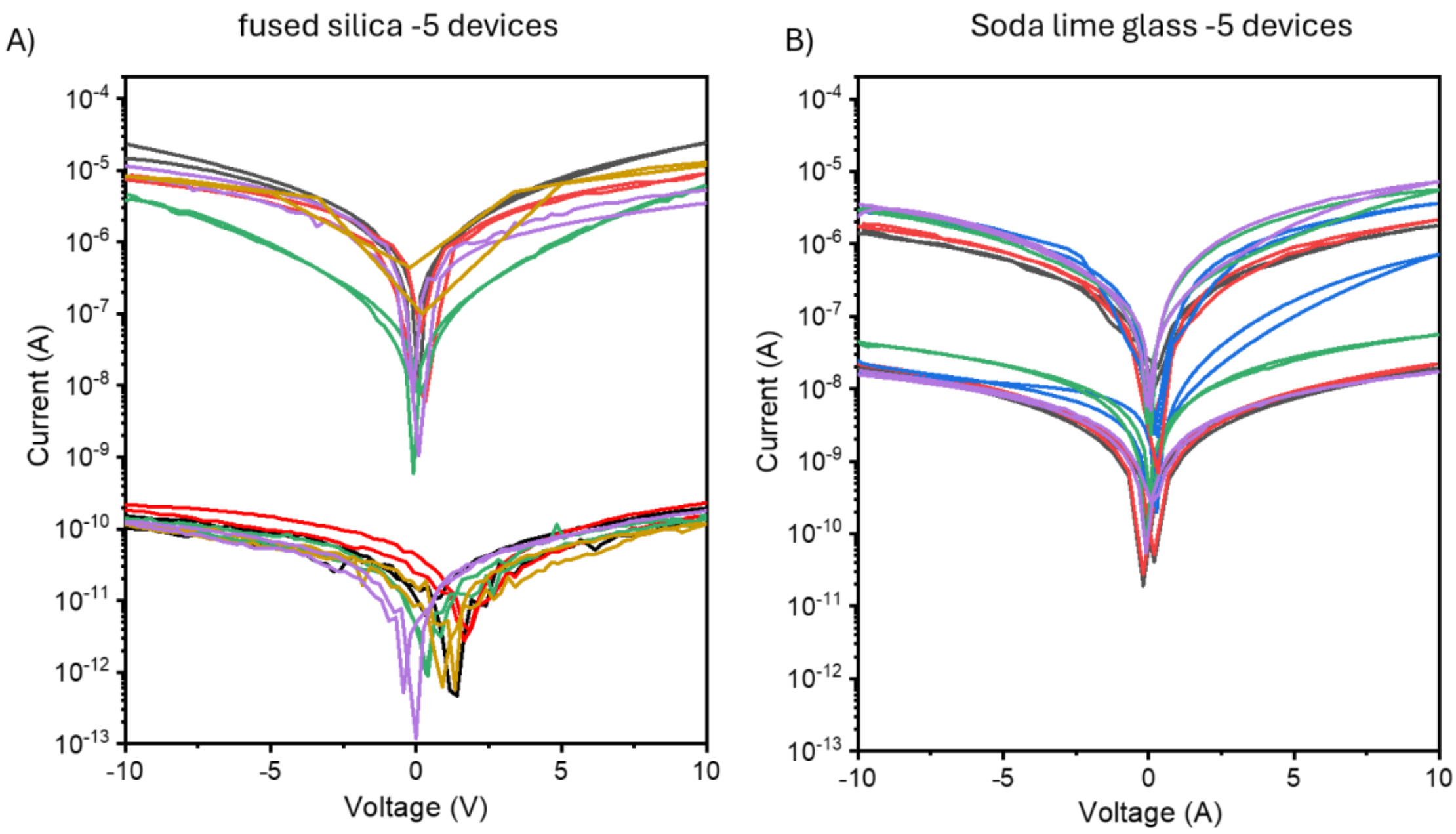

C)

Sample	Responsivity (A/W)	Detectivity (Jones)
Purple	$1,28*10^{-3}$	$6,11*10^{10}$
Green	$4,20*10^{-4}$	$2,02*10^{10}$
Red	$9,31*10^{-4}$	$3,71*10^{10}$
Yellow	$1,01*10^{-3}$	$4,96*10^{10}$
Black	$1,60*10^{-3}$	$8,38*10^{10}$

Figure S11. Current–voltage (I–V) characteristics of five independently fabricated $CsPbBr_3$ photodetectors deposited on (A) fused silica and (B) soda-lime glass substrates, demonstrating the device-to-device reproducibility and substrate-dependent electrical behaviour. (C) Corresponding extracted device figures of merit.

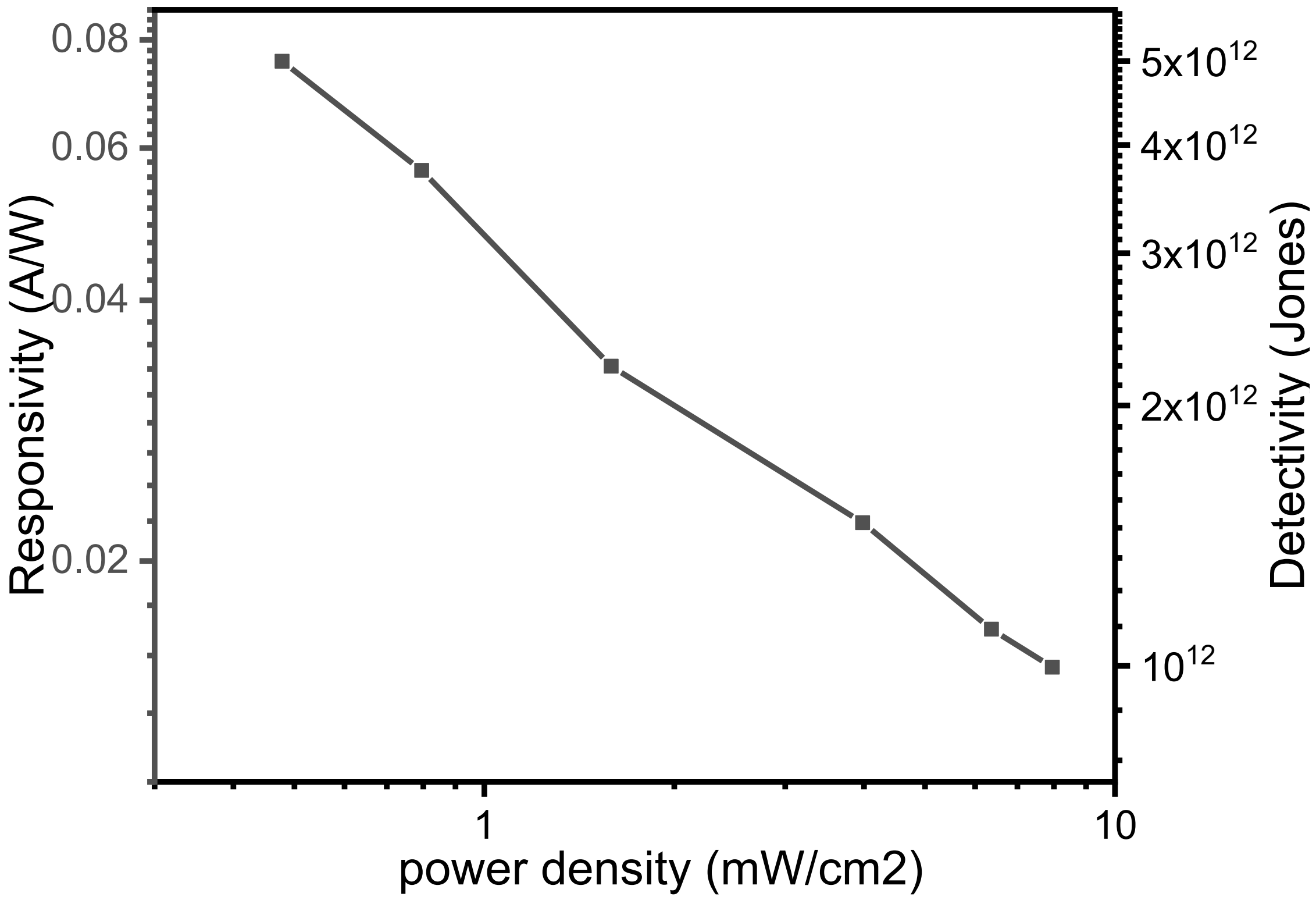

Figure S12. Responsivity (R) and specific detectivity (D) as a function of illumination power density, measured under 488 nm illumination and using a 10V bias.*

Table S3. Device figure of merit.

Materials and device	Preparation method	Measurement conditions	Rise and decay time (ms)	Responsivity (A/W)	Detectivity (Jones)	Ref
Au/$CsPbBr_3$/SmB_6	CVD	4V, 514 nm, 0.5 mW/cm^2	42/24	4.53	$3.36*10^{11}$	[2]
Au/$CsPbBr_3$/Au	CVD	4V, 514 nm, 0.5 $mW//cm^2$	176/80	0.029	$2.6*10^{10}$	[2]
Ag/$CsPbBr_3$/Ag	CVD	3V, 405 nm, 22.3 $\mu W/cm^2$	241/205	506.32	$8.29*10^{12}$	[3]
Au/$CsPbBr_3$/Au	CVD	365 nm, 10 $\mu W/cm^2$	0.5/3.2	0.6	$1.6*10^{10}$	[4]

Au/$CsPbBr_3$/Au Plasma processed	CVD	365 nm, 10 μW/cm^2	0.1/0.3	62.7	7.7*10^{12}	4
Ag/$CsPbBr_3$ (gold catalysed)/Ag	CVD	2V, 450 nm, 6.46 mW//cm^2	17.7/16.5	52.68	18.9*10^{12}	5
Au/$CsPbBr_3$/Au	CVD	2V, 450 nm, 107 mW/cm^2	120/330	0.19	1.85*10^{13}	6
Graphene/$CsPbBr_3$/ Graphene	CVD	2V, 450 nm, 107 mW/cm^2	8.9/12	6.9	1.4*10^{14}	6
Au/$CsPbBr_3$/Ag	PVD	8 V, 0.8 mW/cm^2	/	~ 4,5*10^{-5}	~5*10^{8}	7
Au/$CsPbBr_3$/Au	PVD	8 V, 0.8 mW/cm^2	/	~ 8*10^{-5}	~6*10^{8}	7
Au/$CsPbBr_3$/Au	PVD	5V, 400 nm, 3.83 mW/cm^2	275/550	7.66	4.05*10^{12}	8
Ag/$CsPbBr_3$/Ag	Solution	1V, 520 nm, 27.78 mW/cm^2	3.28/3.32	2.36	6.17*10^{12}	9
SnO_2/$CsPbBr_3$/PTAA/Au	Solution	500 nm, 10^{-4}	0.4/0.43	0.3	10*10^{12}	10
Au/$CsPbBr_3$/Au	Solution	1.5V, 442 nm, 10.8 mW/cm^2	0.6/0.9	34	7.5*10^{12}	11
$CsPbBr_3$	Solution (single platelet)	White light, 4.7 W/cm^2	0.0025/0.0028	~0.12	~1,93*10^{8}	12
$CsPbBr_3$/BN	Solution (nanosheet)	6V, 520 nm, 1 mW/cm^2	152/18	0.01056	1.96*10^{9}	13
ITO/ZnO/$CsPbBr_3$/PTAA/Au	Solution	409 nm	0.01/0.027	0.4	7.01*10^{12}	14
Au/$CsPbBr_3$/Pt	SC	White light, 10 mW/cm^2	230/60	0.028	1.70*10^{11}	15
Au/$CsPbBr_3$/Au	SC	10V, 530 nm, 0.11 μW/cm^2	0.00284/0.00638	36.4	1.59*10^{13}	16
Au/$CsPbBr_3$/Au	SC	5V, 450 nm, 1mW/cm^2	/	0.028	1.8*10^{11}	17

Au/$CsPbBr_3$/Au	SC	10V, 532, 1mW/cm^2	/	5.83	2.5*10^{12}	[18]
Au/$CsPbBr_3$/Au	SC	2V, 530 nm, 216 μW/cm^2	0.4/9	2.5	1.4*10^{13}	[19]
Au/$CsPbBr_3$/Au	CVD	10V, white light ,100 mW/cm^2	/	1.28*10^{-3}	6.11*10^{10}	This work
Au/$CsPbBr_3$/Au	CVD	10V, 405 nm ,40 μW/cm^2	230/361	0.176	8.40*10^{12}	This work

*SC single crystal

Table S4. Comparison of characterization scope between representative CVD-grown $CsPbBr_3$ photodetector studies and the present work.

Study	Growth method	Device architecture	Grain size analysis	Carrier lifetime (TRPL)	Carrier mobility	Noise (NSPD)	X-ray response	Ambient Stability	Generality check
This work	CVD	Lateral, interdigitated Au	13 μm (soda-lime), 19 μm (fused silica)	296 ns	THz-OPTP, Drude-Smith fitting, ~365 cm2 V-1 s-1	3.2×10^{-13} A $Hz^{-1}/^{2}$ at 100 Hz	Demonstrated, sensitivity up to 7.51 μC Gy-1cm-2	1 yr, unencapsulated,	$Cs_2AgBiBr_6$
Liu et al., J. Mater. Chem. C 2024, 12, 17395	APCVD	Au/$CsPbBr_3$/SmB_6 (single-crystal microsheet)	–	2.56 ns (junction) / 3.6 ns (pristine)	FET, 0.4 cm2 V-1 s-1	–	–	20-cycle test	–
Perumalveeramalai et al., Chem. Eng. J. 2024, 492, 152213	CVD	Ag/$CsPbBr_3$/Ag	800 nm	–	SCLC, 0.00241 cm2 V-1 s-1	–	–	90 d	–
Lin et al., ACS Appl. Electron. Mater. 2025, 7, 5404	CVD	Au/$CsPbBr_3$/Au	4.0 μm (15 sccm NH_3) vs 2.0 μm (untreated)	36.6 ns (NH3-treated) vs 4.5 ns (untreated)	SCLC, 0.5 cm2 V-1 s-1 (NH3-treated) vs 0.25 cm2 V-1 s-1 (untreated)	–	–	–	–
Sun et al., ACS Nano 2026, 20, 12954	CVD	Graphene/$CsPbBr_3$/Graphene	–	–	–	–	–	–	$CsPbCl_3$
Guo et al., J. Alloys Compd. 2026, 1068, 188527	CVD	Ag/$CsPbBr_3$ nanowire/Ag	–	–	–	–	–	–	–

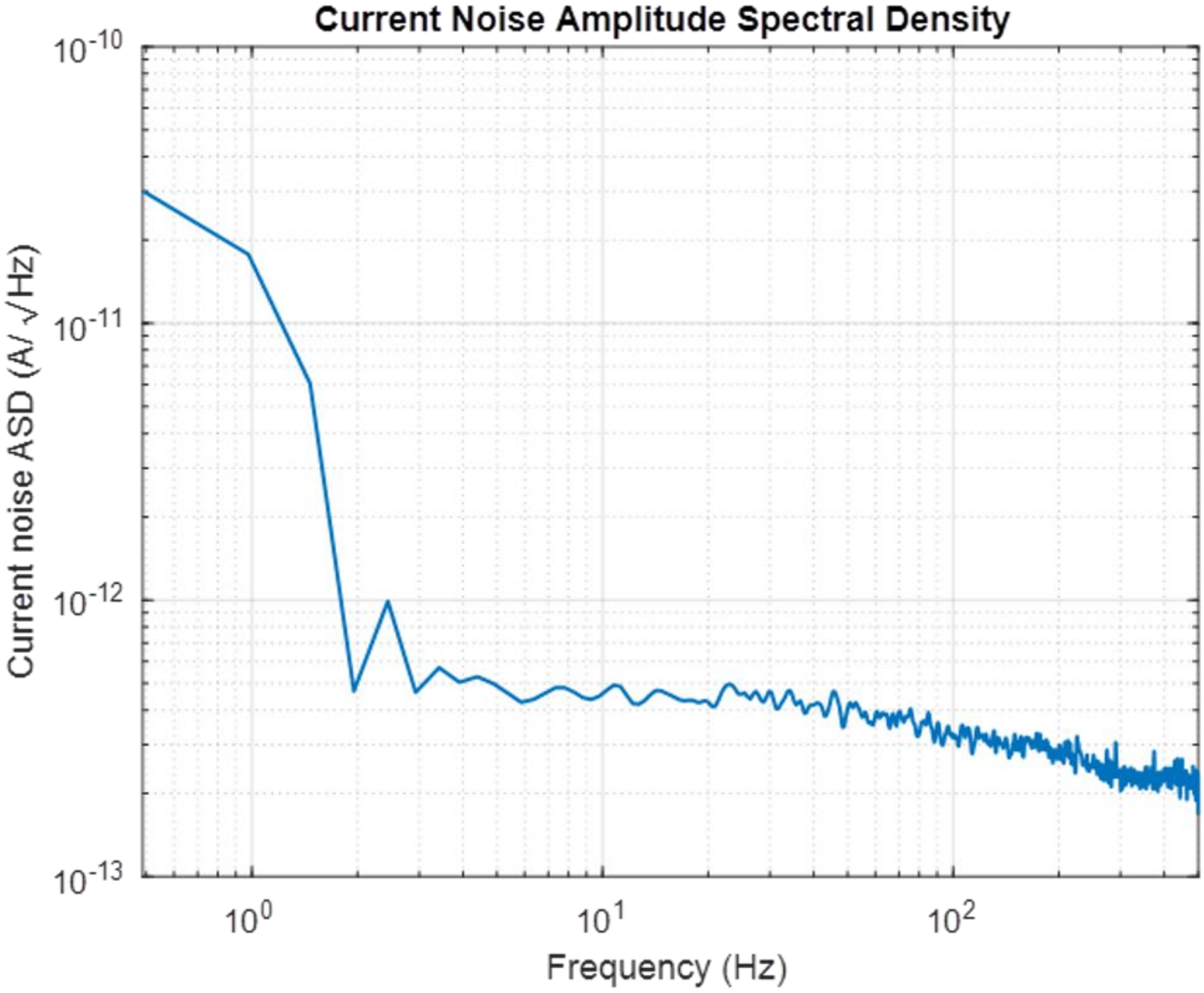

Figure S13. Noise spectral density of the films grown on fused silica.

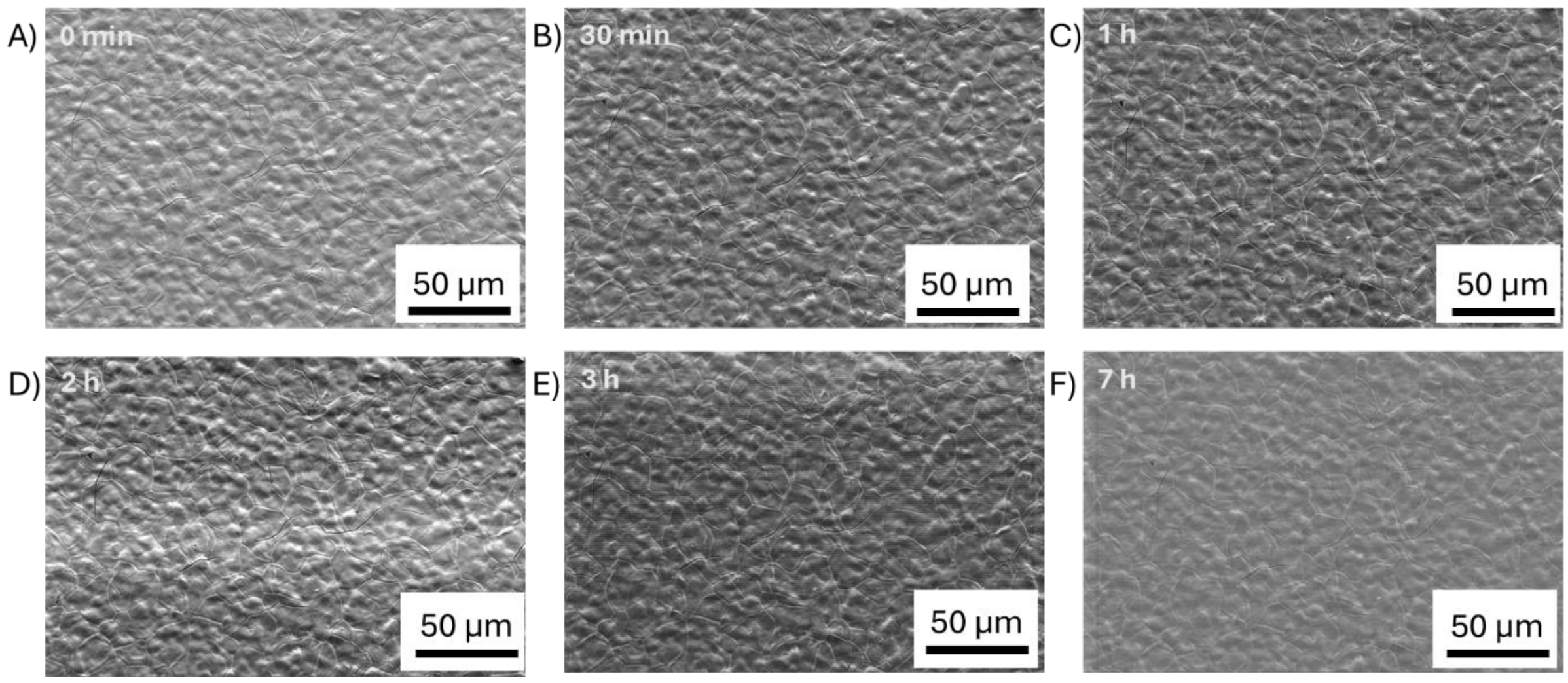

Figure S14. SEM images of the CVD-grown $CsPbBr_3$ film on fused silica recorded during long-term in-situ stability testing under continuous bias (5 V) and illumination (40 mW). Images taken at (A) 0 min, (B) 30 min, (C) 1 h, (D) 2 h, (E) 3 h, and (F) 7 h show no morphological degradation, indicating excellent structural stability of the CVD film.

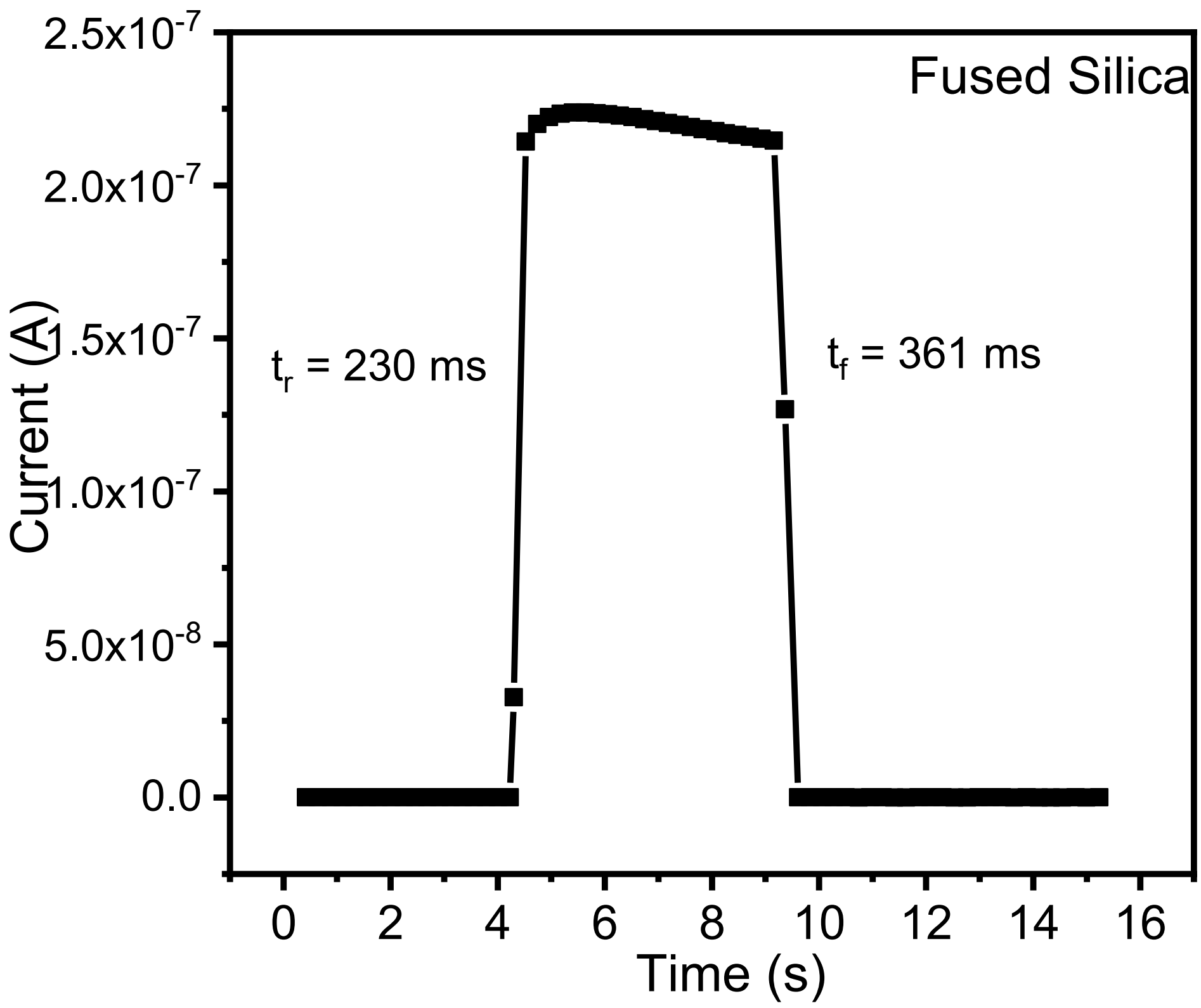

Figure S15. Response time of the CsPbBr3 photodetector deposited by CVD on fused silica.

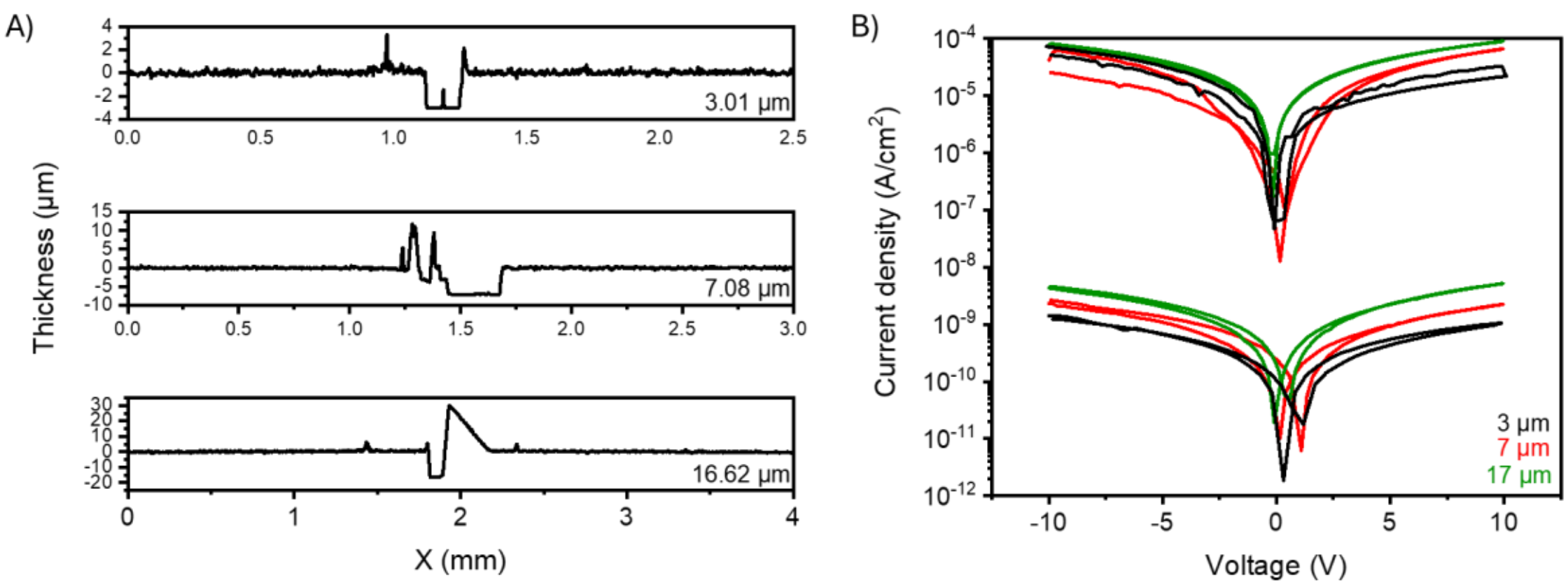

Figure S16. Thickness dependent study: (A) Profilometer data. (B) Current–voltage (I–V) curves of CVD-grown films on fused silica to highlight the influence of thickness on the photo response.

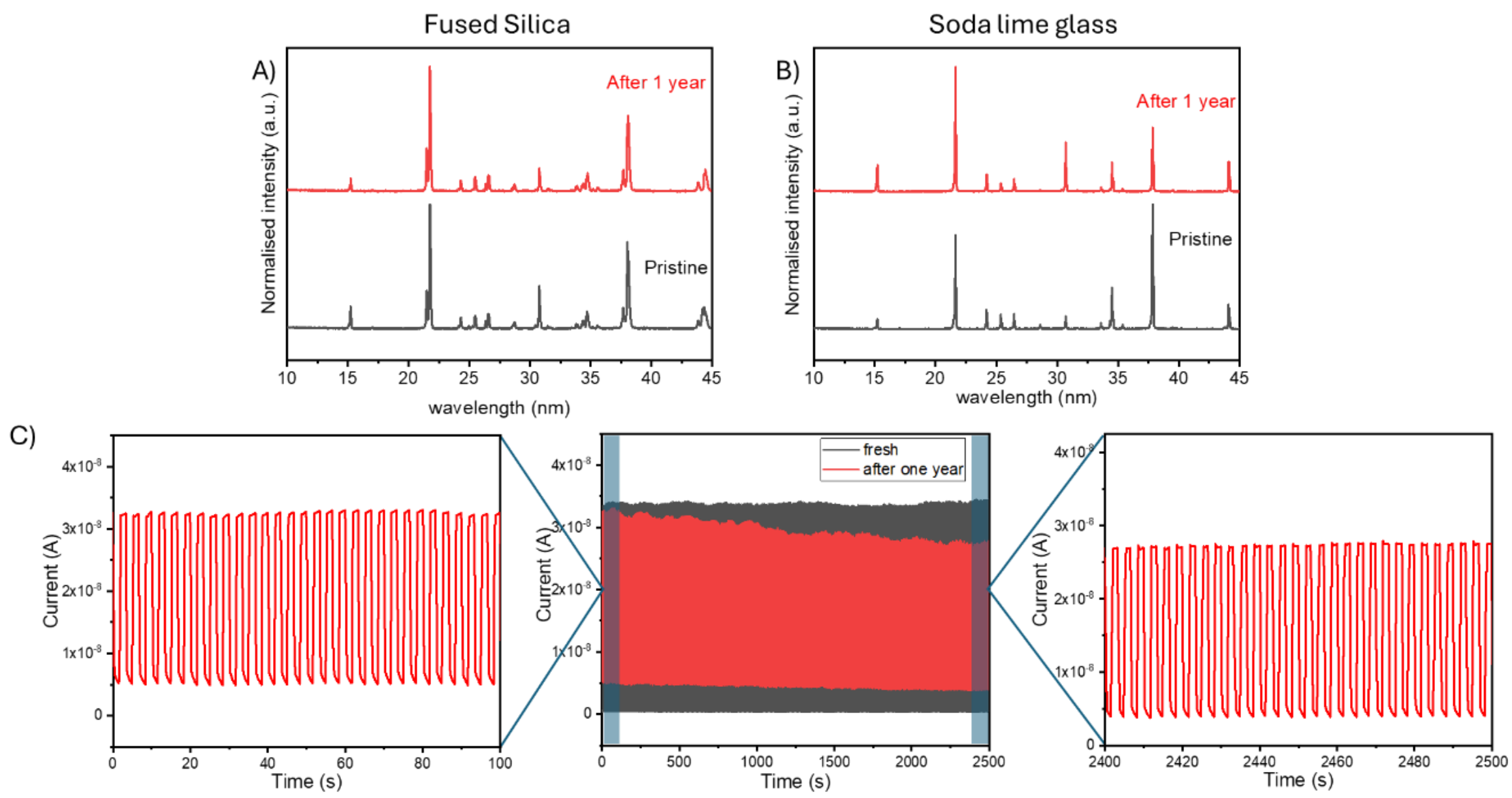

Figure S17. XRD analysis of the aging of CVD-grown $CsPbBr_3$ films stored under ambient laboratory conditions for one year (room temperature, ~50% relative humidity, no encapsulation). (A) $CsPbBr_3$ film on fused silica and (B) $CsPbBr_3$ film on soda-lime glass exhibit identical diffraction profiles after one year, with no emergence of secondary phases. (C) Long-term stability tests after one year. CVD grown film on fused silica under 5V and at 40 µW/cm^2, 405 nm light source. Centre panel = full region , left and right panels =first and last 100 seconds I-t.

$Cs_2AgBiBr_6$ for synthesis robustness of the CVD

To assess the broader applicability and robustness of the developed CVD strategy beyond $CsPbBr_3$, we extended the process to the synthesis of the lead-free double perovskite $Cs_2AgBiBr_6$, a chemically and structurally more complex system requiring precise stoichiometric control over three precursor materials. By appropriately tuning the precursor configuration and growth parameters while maintaining the same reactor geometry and carrier-gas conditions, phase-pure $Cs_2AgBiBr_6$ thin films were reproducibly obtained.

XRD patterns (Figure S18.D) confirm the formation of the double-perovskite phase, with all reflections matching the reference $Cs_2AgBiBr_6$ structure and no detectable secondary phases, indicating effective compositional control during vapor-phase growth. The relatively narrow diffraction peak widths further suggest good crystalline quality, comparable to that achieved for $CsPbBr_3$ under analogous conditions. SEM images (Figure S18.B,G) reveal dense, continuous films composed of compact grains with lateral dimensions on the order of ~2 µm, full substrate coverage and a thickness of +/- 500 nm. Despite the increased chemical complexity of $Cs_2AgBiBr_6$, the films exhibit uniform morphology and well-defined grain boundaries. Raman spectroscopy (Figure S10.E) further corroborates phase purity, with vibrational modes characteristic of the $[AgBr_6]^{5-}$ and $[BiBr_6]^{3-}$ octahedra clearly resolved and no signatures of parasitic bromide or binary phases. Together with the optical data, these results demonstrate that the CVD process preserves high structural and optoelectronic quality when translated to a multinary perovskite system.

The optical quality of the $Cs_2AgBiBr_6$ films was evaluated using steady-state and time-resolved spectroscopy. UV–vis absorption spectra (Figure S18.F) exhibit a well-defined absorption onset consistent with the indirect bandgap characteristic of $Cs_2AgBiBr_6$, while steady-state PL shows the expected broad emission centered at ~645 nm (1.92 eV). The CVD-grown $Cs_2AgBiBr_6$ exhibits a longer effective scattering time obtained from Drude–Smith fitting, which is directly related to carrier mobility. At a pump–probe delay of 2.4 ps, the effective scattering time is approximately 27 fs, significantly larger than that reported for the spin-coated film[20] (16 fs in the reference). The increased effective scattering time mainly originates from a C parameter that is closer to zero, indicating reduced carrier backscattering. This observation is consistent with the results obtained for $CsPbBr_3$, supporting the conclusion that improved grain quality and reduced grain-boundary effects lead to more Drude-like carrier transport in the CVD-grown films.

Importantly, the successful growth of $Cs_2AgBiBr_6$ highlights the generality of the CVD approach, showing that high-quality perovskite films can be achieved across different compositions by parameter tuning rather than process redesign. This compositional transferability, combined with the demonstrated control over $CsPbBr_3$, underscores the versatility of CVD as a amenable to scale-up platform for next-generation perovskite optoelectronic materials.

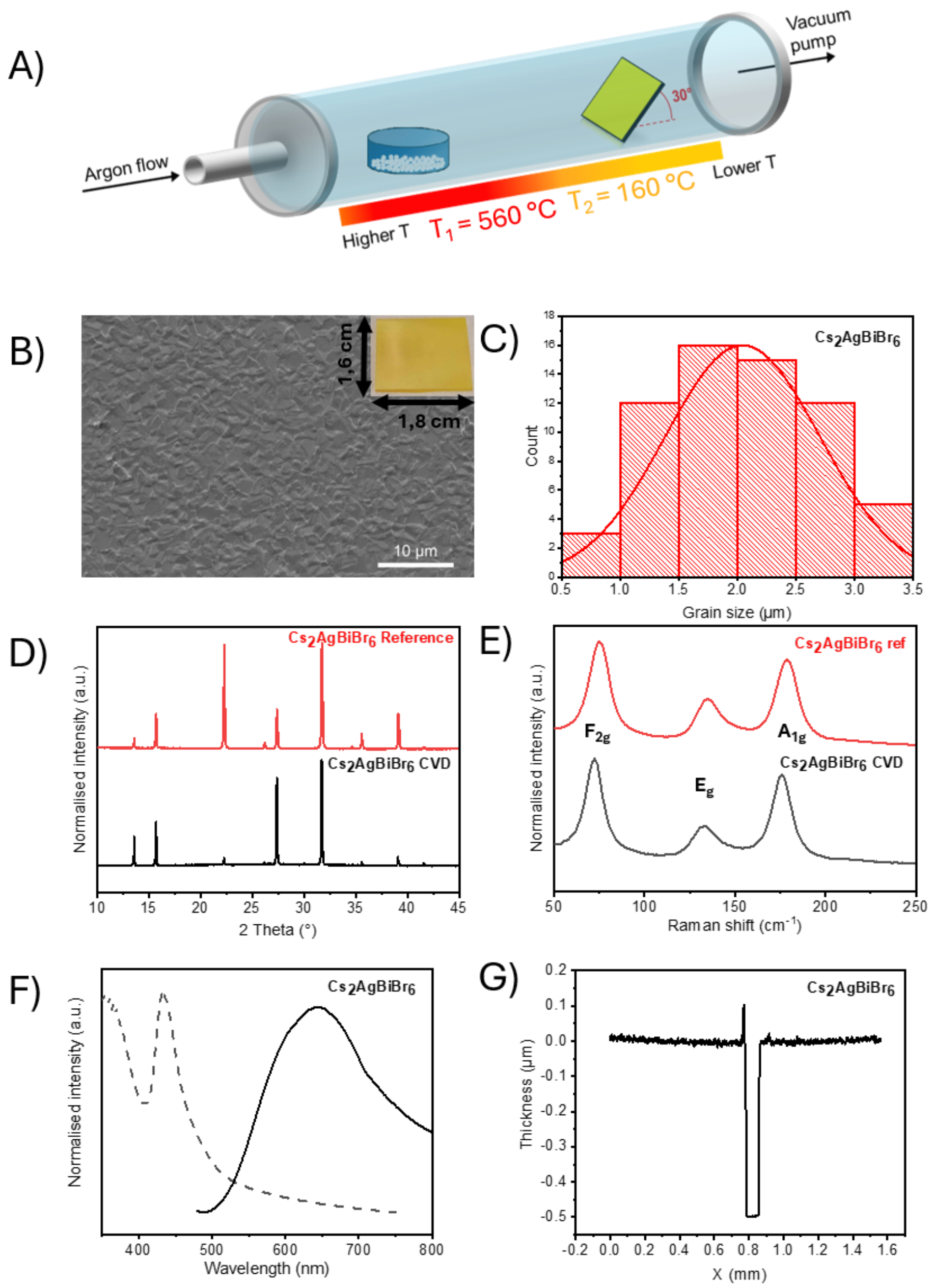

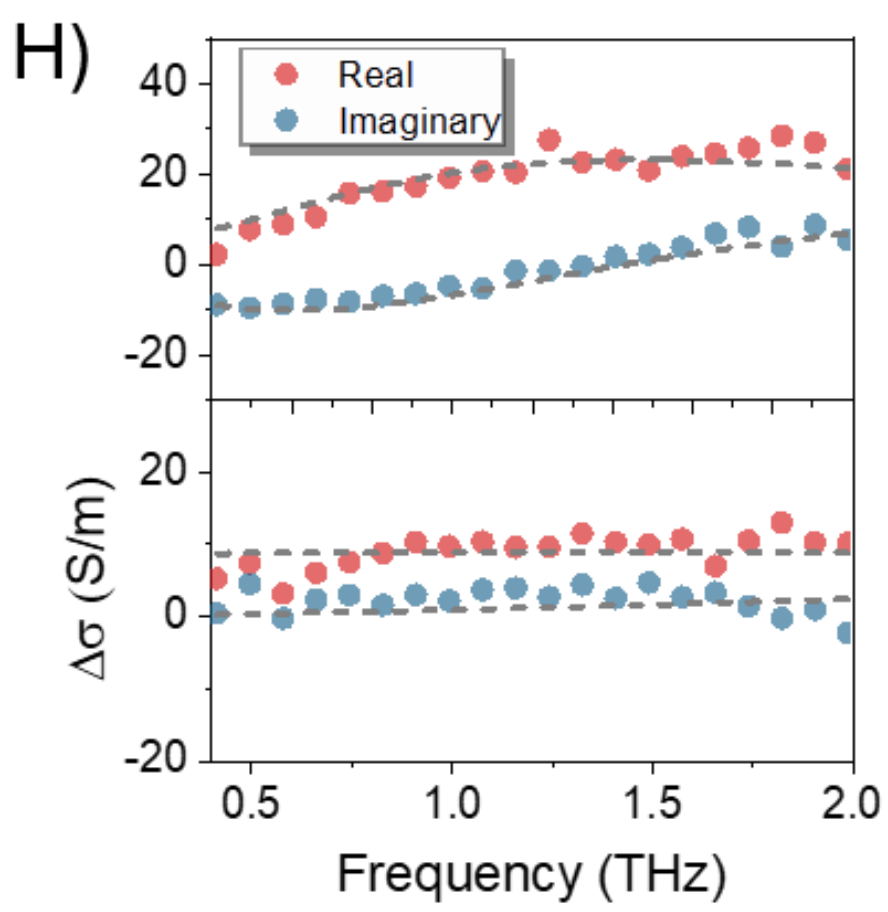

I)

Effective scattering time (τ*(1+c))	0.8ps	2.4ps
Spin-coated	150fs, c~-1	~16 (45fs, c~-0.65)
CVD	5.25 (105fs, c~-0.95)	27 (46fs, c~-0.41)

Figure S18. (A) Schematic of CVD protocol for $Cs_2AgBiBr_6$. (B) SEM image. (C) Grain size distribution. (D) XRD. (E) Raman spectroscopy. (F) UV-vis and PL. (G) Thickness profile. (H) Complex photoconductivity of $Cs_2AgBiBr_6$ grown by CVD growth (fused silica).

References

(1) Vandenwijngaerden, J.; Pradhan, B.; Van Hout, B.; Fron, E.; Araki, Y.; Zhang, X.; Shibata, Y.; Santantonio, D.; Bresoli-Obach, R.; Nonell, S.; Yuan, H.; Xu, J.; Van der Auweraer, M.; Roeffaers, M.; Hofkens, J.; Fukumura, H.; Debroye, E. NIR Luminescence from Deep-Level Traps in CsPbBr3 Microcrystals. *J. Phys. Chem. Lett.* **2025**, *16* (14), 3491–3500. https://doi.org/10.1021/acs.jpclett.5c00545.

(2) Liu, Z.; Cao, G.; Guan, Z.; Tian, Y.; Liu, J.; Chen, J.; Deng, S.; Liu, F. An Advanced Self-Powered Visible-Light Photodetector Based on the Asymmetric Au/CsPbBr3/SmB6 Junction. *J. Mater. Chem. C* **2024**, *12* (43), 17395–17402. https://doi.org/10.1039/D4TC03510K.

(3) Perumalveeramalai, C.; Zheng, J.; Wang, Y.; Guo, H.; Pammi, S. V. N.; Mudike, R.; Li, C. Monolithically Grown CSPbBr3 by Chemical Vapor Deposition for Self-Powered Photodetector. *Chem. Eng. J.* **2024**, *492*, 152213. https://doi.org/10.1016/j.cej.2024.152213.

(4) Lin, Z.; Lin, Z.; Song, J.; Huang, R.; Wu, H.; Chen, K.; Xia, L.; Zhang, Y.; Li, H.; Guo, Y.; Lin, H.; Chu, P. K. High-Performance Ammonia Plasma-Processed CsPbBr3 Perovskite Photodetectors. *ACS Appl. Electron. Mater.* **2025**, *7* (12), 5404–5411. https://doi.org/10.1021/acsaelm.5c00374.

(5) Guo, P.; Zhang, W.; Lv, Q.; Hu, S.; Li, X.; Xu, Z.; Li, L.; Shan, Z.; Yu, K. M.; Ho, J. C.; Xiao, L. CsPbBr3 Nanowires Based Photodetectors for Encrypted Communication. *J. Alloys Compd.* **2026**, *1068*, 188527. https://doi.org/10.1016/j.jallcom.2026.188527.

(6) Sun, X.; Li, S.; Zhang, J.; Gao, W.; Niu, K.; Zhang, L.; Li, Z.; Xie, B.; Wang, Y.; Liu, Y. Enhanced Photodetection of Monocrystalline CsPbBr3 Photoconductor Enabled by Largely Variable Contact Resistance of Graphene Electrodes. *ACS Nano* **2026**, *20* (17), 12954–12965. https://doi.org/10.1021/acsnano.5c21884.

(7) Torres, O. G.; Gordillo, G.; Rossi, I. M.; Roa-Rojas, J. CsPbBr3-Based Photodetector with a Multilayer Architecture and Asymmetric Metal Electrodes. *Mater. Sci. Semicond. Process.* **2025**, *185*, 108898. https://doi.org/10.1016/j.mssp.2024.108898.

(8) Tong, G.; Jiang, M.; Son, D.-Y.; Ono, L. K.; Qi, Y. 2D Derivative Phase Induced Growth of 3D All Inorganic Perovskite Micro–Nanowire Array Based Photodetectors. *Adv. Funct. Mater.* **2020**, *30* (34), 2002526. https://doi.org/10.1002/adfm.202002526.

(9) Yu, M.; Zhang, D.; Xu, Y.; Lin, J.; Yu, C.; Fang, Y.; Liu, Z.; Guo, Z.; Tang, C.; Huang, Y. Surface Ligand Engineering of CsPbBr3 Perovskite Nanowires for High-Performance Photodetectors. *J. Colloid Interface Sci.* **2022**, *608*, 2367–2376. https://doi.org/10.1016/j.jcis.2021.10.141.

(10) Zhou, H.; Song, Z.; Grice, C. R.; Chen, C.; Zhang, J.; Zhu, Y.; Liu, R.; Wang, H.; Yan, Y. Self-Powered CsPbBr3 Nanowire Photodetector with a Vertical Structure. *Nano Energy* **2018**, *53*, 880–886. https://doi.org/10.1016/j.nanoen.2018.09.040.

(11) Liu, X.; Yu, D.; Cao, F.; Li, X.; Ji, J.; Chen, J.; Song, X.; Zeng, H. Low-Voltage Photodetectors with High Responsivity Based on Solution-Processed Micrometer-Scale All-Inorganic Perovskite Nanoplatelets. *Small* **2017**, *13* (25), 1700364. https://doi.org/10.1002/smll.201700364.

(12) Kim, D. B.; Han, J.; Jung, Y. S.; Park, K. S.; Park, Y.; Heo, J.; Cho, Y. S. Origin of the Anisotropic-Strain-Driven Photoresponse Enhancement in Inorganic Halide-Based Self-Powered Flexible Photodetectors. *Mater. Horiz.* **2022**, *9* (4), 1207–1215. https://doi.org/10.1039/D1MH02055B.

(13) Shuang, S.; Lin, J.; Zhang, S.; Yu, C.; Tang, C.; Huang, Y. High-Performance CsPbBr3 Photodetector Enabled by BN Nanosheets via Defect Passivation and Optical Modulation. *ACS Appl. Nano Mater.* **2026**, *9* (21), 9615–9622. https://doi.org/10.1021/acsanm.6c00627.

(14) Luo, M.; Yan, S.; Wei, C.; Xue, L.; Liu, C.; Fayemi, O. E.; Bae, B. S.; Shafie, S. B.; Xiao, M.; Li, Q.; Zhao, Z.; Wu, J.; Lei, W.; Zhang, X. Engineering the Morphology and Component via Multistep Deposition of CsPbBr3 Films toward High Detectivity and Stable Self-Powered Photodetectors. *Adv. Mater. Interfaces* **2022**, *9* (16), 2200450. https://doi.org/10.1002/admi.202200450.

(15) Saidaminov, M. I.; Haque, M. A.; Almutlaq, J.; Sarmah, S.; Miao, X.-H.; Begum, R.; Zhumekenov, A. A.; Dursun, I.; Cho, N.; Murali, B.; Mohammed, O. F.; Wu, T.; Bakr, O. M. Inorganic Lead Halide Perovskite Single Crystals: Phase-Selective Low-Temperature Growth, Carrier Transport Properties, and Self-Powered Photodetection. *Adv. Opt. Mater.* **2017**, *5* (2), 1600704. https://doi.org/10.1002/adom.201600704.

(16) Zhao, X.; Wang, S.; Zhuge, F.; Zhu, N.; Song, Y.; Fu, M.; Deng, Z.; Fang, X.; Meng, G. Nucleation-Controlled Growth of High-Quality CsPbBr3 Single Crystals for Ultrasensitive Weak-Light Photodetectors. *J. Mater. Chem. C* **2023**, *11* (25), 8533–8540. https://doi.org/10.1039/D3TC00550J.

(17) Ding, J.; Du, S.; Zuo, Z.; Zhao, Y.; Cui, H.; Zhan, X. High Detectivity and Rapid Response in Perovskite CsPbBr3 Single-Crystal Photodetector. *J. Phys. Chem. C* **2017**, *121* (9), 4917–4923. https://doi.org/10.1021/acs.jpcc.7b01171.

(18) Zhang, P.; Zhang, G.; Liu, L.; Ju, D.; Zhang, L.; Cheng, K.; Tao, X. Anisotropic Optoelectronic Properties of Melt-Grown Bulk CsPbBr3 Single Crystal. *J. Phys. Chem. Lett.* **2018**, *9* (17), 5040–5046. https://doi.org/10.1021/acs.jpclett.8b01945.

(19) Yang, Z.; Xu, Q.; Wang, X.; Lu, J.; Wang, H.; Li, F.; Zhang, L.; Hu, G.; Pan, C. Large and Ultrastable All-Inorganic CsPbBr3 Monocrystalline Films: Low-Temperature Growth and Application for High-Performance Photodetectors. *Adv. Mater.* **2018**, *30* (44), 1802110. https://doi.org/10.1002/adma.201802110.

(20) Zhang, H.; Debroye, E.; Zheng, W.; Fu, S.; Virgilio, L. D.; Kumar, P.; Bonn, M.; Wang, H. I. Highly Mobile Hot Holes in Cs2AgBiBr6 Double Perovskite. *Sci. Adv.* **2021**, *7* (52), eabj9066. https://doi.org/10.1126/sciadv.abj9066.
